# Coevolution of the Ess1-CTD axis in polar fungi suggests a role for phase separation in cold tolerance

**DOI:** 10.1126/sciadv.abq3235

**Published:** 2022-09-07

**Authors:** Ryan J. Palumbo, Nathan McKean, Erinn Leatherman, Kevin E. W. Namitz, Laurie Connell, Aaron Wolfe, Kelsey Moody, Cene Gostinčar, Nina Gunde-Cimerman, Alaji Bah, Steven D. Hanes

**Affiliations:** ^1^ Department of Biochemistry and Molecular Biology, SUNY-Upstate Medical University, Syracuse, NY 13210, USA.; ^2^ School of Marine Sciences and Department of Molecular and Biomedical Sciences, University of Maine, Orono, ME 04469, USA.; ^3^ Ichor Life Sciences Inc., 2651 US Route 11, LaFayette, NY 13084, USA.; ^4^ Lewis School of Health Sciences, Clarkson University, 8 Clarkson Avenue, Potsdam, NY 13699, USA.; ^5^ The BioInspired Institute, Syracuse University, Syracuse, NY 13244, USA.; ^6^ Department of Biology, Biotechnical Faculty, University of Ljubljana, Jamnikarjeva 101, 1000 Ljubljana, Slovenia.

## Abstract

Most of the world’s biodiversity lives in cold (−2° to 4°C) and hypersaline environments. To understand how cells adapt to such conditions, we isolated two key components of the transcription machinery from fungal species that live in extreme polar environments: the Ess1 prolyl isomerase and its target, the carboxy-terminal domain (CTD) of RNA polymerase II. Polar Ess1 enzymes are conserved and functional in the model yeast, *Saccharomyces cerevisiae.* By contrast, polar CTDs diverge from the consensus (YSPTSPS)_26_ and are not fully functional in *S. cerevisiae*. These CTDs retain the critical Ess1 Ser-Pro target motifs, but substitutions at Y1, T4, and S7 profoundly affected their ability to undergo phase separation in vitro and localize in vivo. We propose that environmentally tuned phase separation by the CTD and other intrinsically disordered regions plays an adaptive role in cold tolerance by concentrating enzymes and substrates to overcome energetic barriers to metabolic activity.

## Teaser

Adaptation to extreme environments involves distinct evolutionary pressures on globular versus disordered regions of proteins.

## INTRODUCTION

Microbial life thrives in extreme environments around the globe (*1*). Particularly well suited to life in extremely cold regions are fungal species, which can be isolated from marine, high-altitude, and polar environments, including arctic glaciers and ice sheets and the extreme cold and dry regions found in Antarctica (*2*, *3*). Fungal species capable of growing in cold environments (psychrophilic) are often tolerant of other extreme conditions such as high salt, desiccation, and high ionizing radiation environments (*4*). How these and other organisms survive under such extreme conditions has been a focus of much attention. Several mechanisms have been found including specific amino acid substitutions in cold-adapted enzymes to make them more flexible, changes in membrane lipid composition to increase fluidity, the production of antifreeze molecules that can also function as compatible solutes at low water activity, ice-binding cryoprotectants, and pigments, as well as changes in gene regulation [(*5*), reviewed in (*4*, *6*)].

However, much remains to be understood. For example, while enzymes from cold-adapted organisms function better at colder temperatures than their temperate orthologs, their calculated activities may not be sufficient to support life under real-life extreme conditions (*7*–*10*). Less is known about how multienzyme complexes, including those that regulate core functions such as DNA replication, transcription, and translation, are able to assemble and carry out their activities under substrate-limiting conditions likely at extreme cold temperatures. In addition, a mystery is how intracellular localization and compartmentation that is required for efficient cellular metabolism and transport occurs in an environment where dynamic interactions and protein diffusion rates are slowed by the cold (*11*).

We study eukaryotic transcription focusing on RNA polymerase II (RNAPII) and its regulation by the peptidyl prolyl cis/trans isomerase, Ess1, which induces conformational changes in the C-terminal domain (CTD) (*12*). RNAPII is a large multisubunit enzyme that carries out RNA synthesis and cotranscriptional RNA processing (capping, splicing, and 3′-end formation) by a carefully orchestrated mechanism during which a series of cofactors are sequentially recruited to the holoenzyme via binding to the CTD of the largest subunit (Rpb1) (*13*). In the temperate yeast *Saccharomyces cerevisiae*, the CTD is composed of a heptad sequence, Tyr-Ser-Pro-Thr-Ser-Pro-Ser (YSPTSPS), repeated 26 times, nearly all of which are exact copies of the consensus sequence. The CTD is posttranslationally modified by phosphorylation on Tyr1, Ser2, Thr4, Ser5, and Ser7, with most of the phosphorylation occurring on Ser5, which is associated with transcription initiation, and Ser2, which is associated with transcription elongation. In addition, both Ser-Pro peptide bonds (S_2_-P_3_ and S_5_-P_6_) are modified by prolyl isomerization, which induces conformational changes (*14*). Cis/trans isomerization is reversible and catalyzed by Ess1 in yeast (called Pin1 in humans) (*15*). Together, these posttranslational modifications direct the orderly recruitment of cofactors to promote efficient transcription and RNA processing (*16*). The pattern of modifications is referred to as the “CTD code” (*17*).

The CTD, which is intrinsically disordered, not only helps coordinate individual steps in the RNAPII transcription cycle but also is proposed to nucleate a process known as liquid-liquid phase separation (LLPS) (*18*). LLPS is highly temperature and salt dependent (*19*), and the formation of macromolecular condensates through phase separation helps regulate enzyme activity (*20*). The CTD is thought to concentrate, via phase separation, RNAPII along with DNA regulatory elements (enhancers/promotors) and associated proteins into dense “transcription factories” (*21*). These factories can be visualized as subcellular foci within nuclei where transcription occurs (*22*). Key to the present study is that phase separation by the CTD is affected by temperature and salt conditions, and as we will show is highly dependent on amino acid sequence.

Binding of RNAPII cofactors to the CTD is dependent on conformational isomerization: Some proteins bind the cis form, while others prefer the trans form (*23*). Thus, Ess1 is crucial for efficient CTD function, and so far, all eukaryotic organisms that have an RNAPII-CTD also have an Ess1 ortholog (or related prolyl isomerase), suggesting that they coevolved (*12*). Ess1 is also an attractive model for studying cold adaptation because the enzyme is small, easily purified, and highly conserved in eukaryotes, and the structures of Ess1 from *S. cerevisiae* and *Candida albicans* as well as human Pin1 have been determined by high-resolution x-ray crystallography (*24*–*26*). Detailed information is also available about how Ess1/Pin1 binds its CTD substrate (*25*, *26*).

Here, we established the fungal Ess1-CTD axis as a platform to study cold adaptation of transcription at the genetic, molecular, and biophysical levels. We found that Ess1 is conserved in polar fungi and identified specific amino acid substitutions that are predicted to increase protein flexibility to enable enzymatic activity in the cold. The Ser-Pro motifs within the CTD, which are known to bind Ess1 (and other CTD-binding proteins), are nearly invariant, suggesting that the Ess1-CTD interaction is maintained through evolution. Unexpectedly, the CTDs from cold- and salt-adapted fungi from diverse geographic environments (including the Arctic and Antarctic) are highly divergent at positions 1, 4, and 7 within the CTD and did not fully substitute in *S. cerevisiae* nor direct proper localization of Rpb1 to yeast nuclei. These divergent CTDs showed distinct LLPS properties in vitro in response to changes in temperature and salt concentration. On the basis of these findings, we propose that localized sequence divergence within the CTD, and potentially in the intrinsically disordered regions (IDRs) of other proteins, may be an evolutionary driver to enable adaptation of organisms to extreme environments by altering biophysical properties such as the ability to undergo phase separation. Moreover, in cold-adapted organisms, phase separation may help concentrate enzymes and their substrates within cells to overcome energetic barriers to metabolic activity.

## RESULTS

### Description of cold-adapted and halophilic fungal species

**Fig. 1.**
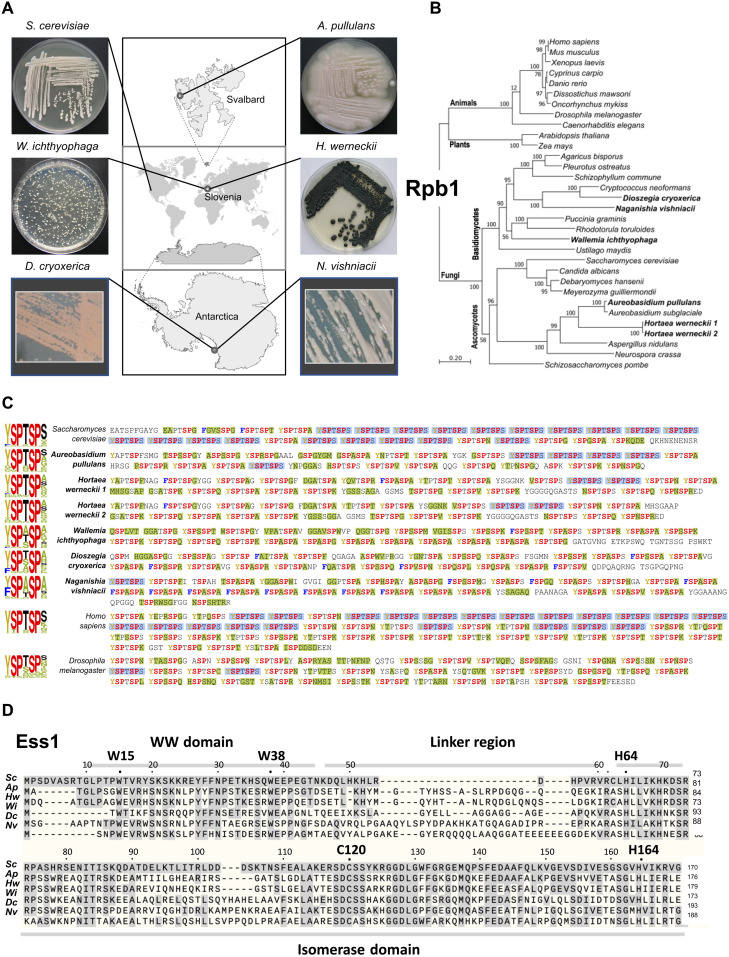
Fungal species and key RNAPII regulators in this study. (**A**) Physical appearance of petri dish–grown cultures and the geographic sites of their isolation. *S. cerevisiae*, *A. pullulans*, *W. ichthyophaga*, and *H. werneckii* were grown on malt extract agar, in case of *W. ichthyophaga* with 25% of added NaCl (w/v), and incubated for 7 days (*S. cerevisiae* and *A. pullulans*) or 14 days (*H. werneckii* and *W. ichthyophaga*) at 24°C. Photos of *D. cryoxerica* and *N. vishniacii* are courtesy of K. Earle (https://mycocosm.jgi.doe.gov/mycocosm/home) (*80*). Additional details and references are in table S1. (**B**) Maximum likelihood phylogenetic analysis of the RNAPII large subunit, Rpb1, after removal of the CTD region from the alignment of Rpb1 amino acid sequences. Polar yeast species and halophilic fungi studied here are indicated in bold lettering. See text for details. (**C**) Sequences of the RNAPII Rpb1 subunit CTDs of species used in this study, annotated according to the conventional heptad repeat nomenclature (Y_1_S_2_P_3_T_4_S_5_P_6_S_7_). Highly conserved residues (SP) are in red, common substitution Y1F is indicated in blue, and other nonconsensus substitutions are highlighted in green. While the number of repeats is similar in the fungal species, the sequences diverge, particularly at positions 1, 4, and 7 in the polar species. Two sequences are shown for *H. werneckii*, which is likely a diploid hybrid. See text for details. Additional CTD alignments and additional species CTDs are shown in figs. S1 and S2, respectively. (**D**) Sequences of predicted proteins from polar yeast species aligned with that of *S. cerevisiae* Ess1 (ScEss1). Signature residues are conserved in all species, including W15 and W38 in ScEss1 that define the WW domain involved in substrate targeting and active-site residues H64, C120, and H164 in ScEss1 that play important roles in catalysis. Note the variable length and sequence of the linker regions that join the WW and isomerase domains.

To investigate mechanisms of transcription in cold- and salt-adapted organisms, we collected fungal species from diverse environments (Fig. 1A). The black yeast *Aureobasidium pullulans* was isolated from subglacial ice of the Kongsvegen glacier on Spitsbergen (Svalbard, Norway) (*27*), although it can be found in diverse environments including Antarctica (*28*). *Hortaea werneckii*, a black yeast typically found in hypersaline evaporates of sea water, was isolated in Slovenia; it is also present in seawater worldwide and, according to molecular data, is also in glacial ice (*29*). *H. werneckii* grows in normal laboratory media as well as media almost saturated with NaCl. The most halophilic fungus known, *Wallemia ichthyophaga*, was originally isolated from fish near the Lofoten Islands, Norway, north of the Arctic Circle (*30*). It is a rare species found in hypersaline environments in temperate regions, and in northern polar regions, and is one of only a few fungi requiring low water activity (increased viscosity) for growth. The isolate used here is from a saltern in Slovenia (*31*). Strictly speaking, *H. werneckii* and *W. ichthyophaga* (which is not technically a yeast) are halophiles (*31*, *32*), but because they also live in extreme cold environments including polar regions, we included them in our set of species that we refer to as “cold-tolerant,” “polar,” or “extremophile” yeast. *Dioszegia cryoxerica* and *Naganishia vishniacii* were isolated from soil samples collected in the Taylor and Wright valleys, respectively, in the McMurdo Sound region of South Victoria Land, Antarctica (*33*, *34*) and have yet to be isolated outside of Antarctica. Additional information about these species is found in table S1.

These representative psychrophilic fungal species were chosen because they were isolated from different hemispheres, are culturable, and have genomic sequence available. Because of their geographic isolation and distinct phylogenetic origin, we reasoned that cold (and salt) adaptation by these organisms would have evolved independently, potentially revealing both common and distinct genetic and physiological mechanisms for cold tolerance. The model organism *S. cerevisiae* was used as a surrogate host and is considered a mesophilic yeast.

### Identification of Rpb1 and Ess1 orthologs in cold-tolerant yeasts

The Rpb1 subunit of RNAPII is encoded by the *RPO21* (RNA POL 2 subunit 1) gene. We searched the nonredundant protein GenBank and transcriptomic databases using the *S. cerevisiae RPO21*-encoded sequence (Rpb1) as a query. One Rpb1 homolog per fungal species was found, except for the diploid hybrid *H. werneckii*, which had two homologs, and *D. cryoxerica*, which also had two homologs, perhaps indicating a duplication or that it is also a diploid hybrid. There was 55 to 58% identity between *S. cerevisiae* Rpb1 and the other Rpb1 sequences analyzed (after excluding the highly variable and difficult-to-align CTD region). The gene phylogeny derived from Rpb1 shows the relatedness of species used in this study relative to other fungi (Fig. 1B) and was close to the canonical phylogeny of the species with only a few poorly supported branches, e.g., the position of *W. ichthyophaga* is difficult to determine with certainty even in more thorough phylogenetic studies (*32*). Expectedly, the ascomycetes *A. pullulans* and *H. werneckii* group closer to *S. cerevisiae* and *C. albicans* (both are ascomycetes), while the Antarctic yeast *D. cryoxerica* and *N. vishniacii* and the halophile *W. ichthyophaga* group with other basidiomycetous species such as *Cryptococcus neoformans* and *Ustilago maydis.*

Unlike the main body (globular domains) of Rpb1, the critical CTD sequences were markedly different (Fig. 1C). In *S. cerevisiae* and other model yeasts such as *Schizosaccharomyces pombe* and *C. albicans*, the CTD is composed of ~26 nearly identical heptad repeats (YSPTSPS), while in vertebrates such as zebrafish (*Danio rerio*) and humans, the CTD is composed of 52 repeats that are highly conserved in the first half, with variation primarily in the latter half at position 7 of the heptad (*17*, *35*). In contrast, the polar yeast CTDs are highly divergent throughout their lengths, at heptad positions 1, 4, and 7. However, the “core” Ser-Pro motifs within the CTD (S_2_-P_3_ and S_5_-P_6_) remain essentially invariant (see also figs. S1 and S2). We wondered why the CTDs from polar yeasts exhibit such sequence divergence and exactly what effect the variations at positions 1, 4, and 7 would have on Rpb1 function, questions that we addressed below.

Using the *S. cerevisiae Ess1* (ScEss1) protein sequence as query, we searched the GenBank nonredundant protein databases with BLAST. Sequences of Ess1 from *A. pullulans*, *H. werneckii*, *W. ichthyophaga*, *D. cryoxerica*, and *N. vishniacii* were recovered with BLAST from the whole-genome sequences and the corresponding predicted proteomes (GenBank) (*32*–*34*, *36*, *37*). Two orthologs of the *ESS1* gene were found in *H. werneckii*, which is a diploid hybrid (*38*), while in the other species, one ortholog per strain was found. Compared with the ScEss1 enzyme, the sequences are 45% (*A. pullulans*), 42% (*H. werneckii*), 42% (*W. ichthyophaga*), 40% (*D. cryoxerica*), and 37% (*N. vishniacii*) identical at the amino acid level (*39*). For comparison, the identities between ScEss1 and *C. albicans* Ess1 (CaEss1), *Drosophila melanogaster* Dodo, and human Pin1 are 43, 46, and 46%, respectively. The sequence alignments (*40*) predict conservation of key features such as an N-terminal WW domain (involved in protein-protein interactions) joined by a variable linker to a C-terminal prolyl isomerase catalytic domain (Fig. 1D). The signature tryptophan residues (W15 and W38) of the WW domain, which helps bind substrates, are conserved, as are active-site histidines (H64 and H164) and cysteine (C120) within the catalytic domain (*25*). The gene phylogeny using Ess1 was similar to that of Rpb1 (fig. S3); however, the relatively short sequence of Ess1 (170 amino acids) limits its reliability.

### Compromised function of polar CTDs in budding yeast

To test whether the divergent CTDs encoded by polar yeast *RPO21* orthologs were functional in *S. cerevisiae*, we replaced the normal CTD of the *S. cerevisiae* Rpb1 with polar CTD sequences (fig. S4A). We then tested these constructs in a genetic complementation assay (fig. S4B), in which the host cells contain a genomic deletion of *RPO21*, which is essential for growth, but are viable because of a plasmid (2 μM; *URA3*) expressing the *S. cerevisiae* Rpb1 subunit. Cells were transformed with a second plasmid (2 μM; *HIS3*) expressing the chimeric Rpb1-CTD protein and subjected to a plasmid shuffle strategy, and the cured cells were plated and scored for loss of the *URA3*-marked *RPO21* plasmid, indicating complementation levels. The results using ScRpb1-[CTD_polar_] chimeras in cells grown at 18°C showed that the CTDs functioned poorly in vivo, with the Antarctic yeast CTDs not functioning at all under these conditions (Table 1, left).

**Table 1.** Polar CTDs do not fully complement in *S. cerevisiae*. Curing, plating, and scoring were done at the indicated temperature. At least two individual colonies were picked for curing from each of two independent DNA clones expressing a given CTD construct. Cured (%) is defined by cells that were Ura^−^ (lost ScRpb1 plasmid) and His^+^ (retained polar CTD plasmid). Complementation rate is the % cured divided by the positive control percentage (58%) cured × 100 rounded to the nearest whole number.

**Species**	***n***	**Cured (%)**	**Complementation rate**	***n***	**Cured (%)**	**Complementation rate**
**Temperature**	**18°C**			**30°C**		
Vector only (negative control)	450	0	0	150	0	0
*S. cerevisiae* (positive control)	450	58.2	100	225	87	100
*A. pullulans*	441	13.6	23	300	55	63
*H. werneckii 1*	200	35.5	61	300	73	84
*H. werneckii 2*	525	21.5	37	225	62	71
*W. ichthyophaga*	450	0.2	0.3	225	68.7	79
*D. cryoxerica 1*	*	0	0	300	77.7	89
*D. cryoxerica 2*	450	0	0	225	68	78
*N. vishniacii*	450	0	0	300	45	52

*D. cryoxerica 1 was tested at 18°C using 5-fluoroorotic acid (5-FOA) selection. No cured colonies were detected after screening >200,000 cells.

To determine whether genetic changes in the CTD occurred during our experiments, for example, by recombination to generate more consensus-like sequences, we rescued plasmids from cells and determined the DNA sequences of the CTD. We found no changes from the original *A. pullulans* CTD (ApCTD) and *H. werneckii* CTD (HwCTD) sequences, confirming that these CTDs can function to some extent in *S. cerevisiae*. Cells bearing either ApCTD or HwCTD grew at 30°C, prompting us to repeat the curing experiments at 30°C. Much to our surprise, all the polar CTDs complemented well at 30°C (Table 1, right). When these cured cells were tested for growth in liquid media, they grew well at both 18° and 30°C (fig. S5). We do not currently understand why curing occurred at 30°C but not at 18°C, but one possibility is that 18°C represents stress for *S. cerevisiae*, during which time the function of the CTD is known to be especially critical. We suspected therefore that polar CTD function was compromised in *S. cerevisiae* and that this is revealed under stress.

**Fig. 2.**
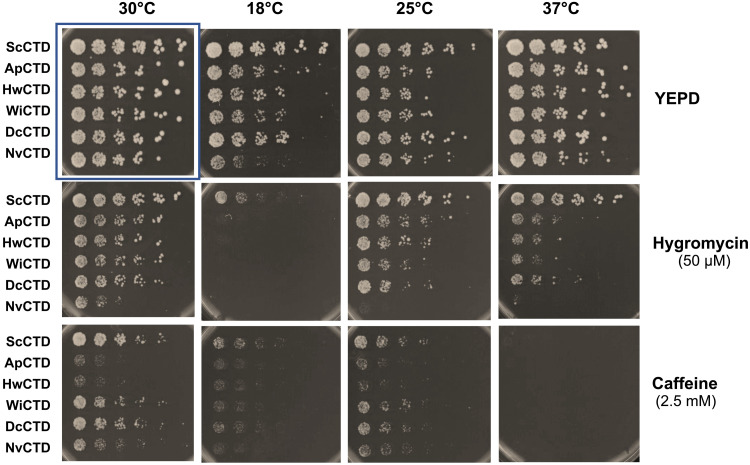
The CTDs from cold-tolerant yeasts do not fully compensate for the *S. cerevisiae* CTD in stress conditions. Serial dilutions (1:3) of *S. cerevisiae* (*rpb1*Δ::*kanMX*) strains bearing plasmids that express Rpb1-CTD_n_ (*CEN HIS3*) from the indicated species were grown on yeast extract peptone dextrose (YEPD) plates at different temperatures with or without the indicated chemical stressors. The strains were derived from CBW1 obtained in curing experiments (Table 2, 30°C); see Materials and Methods for details. Before spotting, cells were grown overnight to saturation in CSM-His liquid medium. After spotting, plates were incubated at 25°, 30°, or 37°C for 2 days or at 18°C for 4 days. The box indicates control non-stress conditions (30°C, no drug).

To test this idea, we subjected cells bearing the ScRpb1-[CTD_polar_] chimeras to a variety of stress conditions. In the examples shown (Fig. 2), serial dilutions of cells were spotted on solid medium to test the effects of temperature and drugs on growth. As with the 18°C curing experiments, the polar CTDs were not as efficient as the native ScCTD in promoting growth, with some polar CTD strains less able to support growth than others. For example, growth of all the polar CTD cells was slower at 18°C and was exacerbated by the presence of hygromycin or caffeine, compared to the control. However, at higher temperatures (25°, 30°, and 37°C), the NvCTD strain was selectively sensitive to hygromycin, whereas ApCTD and HwCTD strains were particularly sensitive to caffeine, especially at 25° and 30°C. Sensitivity to hygromycin and caffeine is often interpreted as suggesting defects in cell wall biogenesis or maintenance (*41*). Ess1 mutants are also sensitive to these drugs (*14*) as expected, given that it works in concert with the CTD. In summary, while the polar CTDs function in a surrogate host under some conditions, they did not provide sufficient activity under all conditions.

### Robust function of polar Ess1s in budding yeast

To test whether the *ESS1* gene sequences identified in polar yeasts encode functional orthologs of the Ess1 enzyme, we cloned and expressed them in a *S. cerevisiae* genetic complementation assay (fig. S4, A and C) analogous to that used for the CTD above (see Materials and Methods). The controls, an empty vector or plasmid expressing the *D. melanogaster ESS1* ortholog (*dodo*), which is known to complement (*42*), showed 0 and 68% curing rates, respectively (Table 2). The polar yeast Ess1s all had high curing rates (46 to 65%), representing complementation rates of 68 to 95% relative to the positive control. This indicates that they are all bona fide Ess1 orthologs and, despite their being isolated from extremophilic fungi, were stable and functional within the cellular milieu of their mesophilic host. The results were not unexpected given that a number of yeast and metazoan Ess1 orthologs also function in this assay (*12*). We suggest that their ability to cross-complement is a result of highly conserved substrate recognition and enzymatic activities as a result of their coevolution with the essentially invariant Ser-Pro motifs within the CTDs from the cold-adapted species, as well as the need to bind other Ser-Pro containing substrates.

**Table 2.** Polar Ess1s complement in *S. cerevisiae*. Curing, plating, and scoring were done at 18°C. At least two individual colonies were picked for curing from each of the two independent DNA clones expressing a given Ess1 protein. For *N. vishniacii*, the “intron” refers to a possible intron identified in the coding sequence (see Materials and Methods). *n*, number of colonies picked, patched, and scored (see Materials and Methods for details). Cured (%) is defined by cells that were Ura^−^ (lost *D. melanogaster dodo* plasmid), Trp^+^ (retained polar Ess1 plasmid), and His^+^ (retained chromosomal deletion; *ess1*Δ::*HIS3*). Complementation rate is the % cured divided by the positive control percentage (68%) × 100 rounded to the nearest whole number.

**Species**	***n***	**Cured (%)**	**Complementation rate**
Vector only (negative control)	147	0	0
*D. melanogaster* (positive control)	300	68.3	100
*A. pullulans*	300	46.3	68
*H. werneckii*.	300	60.0	88
*W. ichthyophaga*	225	54.7	80
*D. cryoxerica*	450	65.1	95
*N. vishniacii* (“intron” retained)	559	61.2	90
*N. vishniacii* (“intron” removed)	600	58.2	85

Note that the curing was done (Table 2) at a low temperature (18°C) for *S. cerevisiae* because we were initially concerned about the stability and/or function of polar Ess1s at a higher temperature. In subsequent experiments, we showed that *S cerevisiae* cells bearing polar Ess1s grow well at both 18° and 30°C and at a high, stress-inducing temperature (37°C) (fig. S6). These results are consistent with earlier studies that show that cold-adapted enzymes are typically active at higher temperatures, so long as they do not reach their threshold for unfolding (*43*). These findings distinguish between the polar Ess1s, which are functional under multiple stress conditions (18° and 37°C), and the polar CTDs, which are fully functional only under certain conditions.

### Comparison of mesophilic and polar Ess1s

**Fig. 3.**
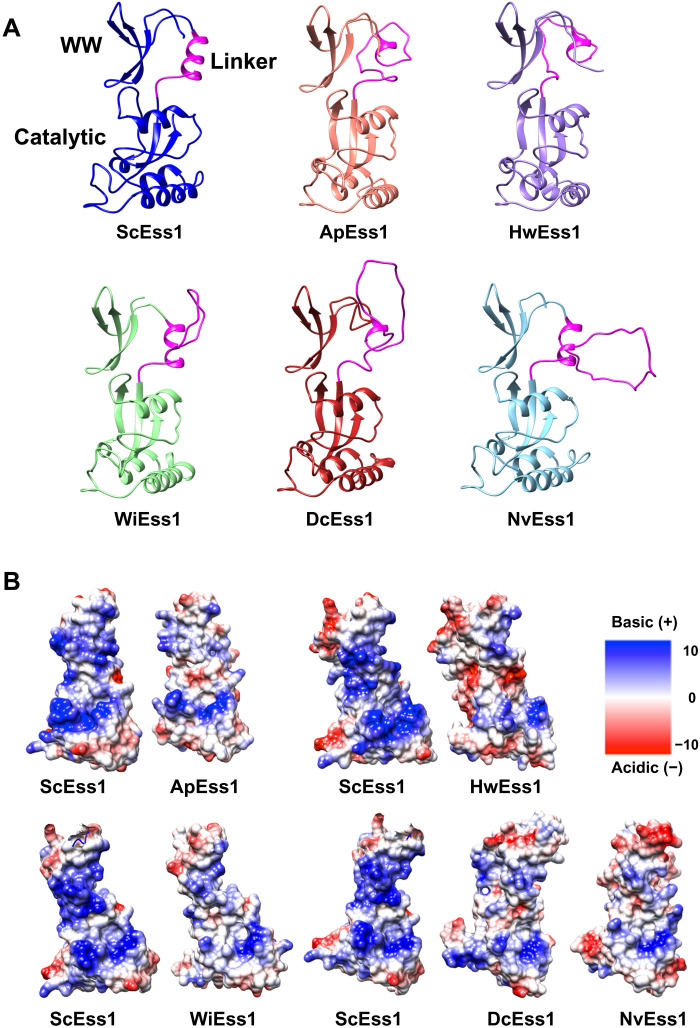
Structural models and surface charge distributions of polar Ess1 enzymes. Models are based on the x-ray crystal structure of ScEss1 [Protein Data Bank (PDB) ID: 7KKF]. (**A**) Ribbon diagrams of Ess1s highlighting the extended linkers joining the globular WW and catalytic domains. ScEss1 (*S. cerevisiae*), ApEss1 (*A. pullulans*), HwEss1 (*H. werneckii*), WiEss1 (*W. ichthyophaga*), DcEss1 (*D. cryoxerica*), and NvEss1 (*N. vishniacii*) are compared. The models do not predict highly structured linkers with an extended α helix as in the ScEss1 structure. (**B**) Surface charge distribution of cold-tolerant Ess1s. Space filling models show side chains of basic residues (Arg and Lys) in blue and acidic residues (Glu and Asp) in red. The models of Ess1 enzymes (as above) are aligned in the same relative orientation as ScEss1 to highlight the decrease in basic charge densities and, in some cases, increased surface acidity as observed for other cold-adapted enzymes. Surface-exposed lysine residues that have been substituted in the cold-tolerant Ess1s are at positions 21, 34, 46, 51, 70, 87, 95, 106, 115, 124, 149, and 167 of ScEss1.

**Fig. 4.**
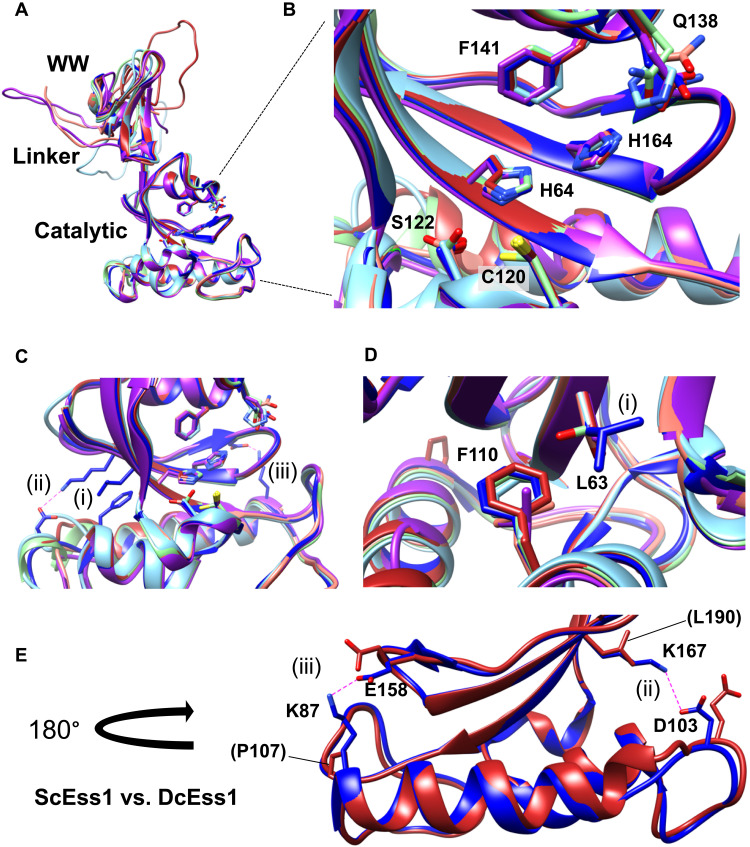
Modeling reveals potential cold adaptations in Ess1. (**A** to **E**) Color scheme is as in fig. S6: ScEss1 (blue), ApEss1 (salmon), HwEss1 (purple), WiEss1 (light green), DcEss1 (red), and NvEss1 (cyan). (A) Superposition of polar Ess1s on the 2.4-Å structure of ScEss1. The globular WW and catalytic (isomerase) domains superimpose very well, but the linkers between them are variable in length and likely unstructured in the polar Ess1s. (B) View of the active site showing highly conserved substrate binding and catalytic residues. Numbering is from the ScEss1. All are identical except for a histidine in NvEss1 instead of glutamine at position 138 in ScEss1 and the other four proteins. (C) Nonpolar interactions (i) and salt bridges (ii and iii) that likely stabilize the hydrophobic core in ScEss1 and support the β-strand structure that forms the active site, respectively. (D) Close-up of two core hydrophobic residues, L63 and F110, in ScEss1. In four of five of the polar Ess1s, L63 is replaced by serine, a polar residue not normally compatible with a hydrophobic core. (E) Comparison of a portion of ScEss1 (blue) and DcEss1 (red) from (C) highlighting the loss of two key salt bridges. The K87P and K167L substitutions (ScEss1 numbering) that occur in the Antarctic protein, DcEss1, would eliminate the K87-E158 and K167-E103 ionic interactions, potentially providing more flexibility in the active-site scaffold.

While the function of the polar Ess1s appears to be highly conserved, we wondered what structural adaptations enable them to function in the cold. We therefore modeled the cold-tolerant Ess1s using the x-ray crystal structure of ScEss1 (*25*). The globular domains are almost identical in structure, as expected given their sequence conservation and functional complementation in *S. cerevisiae* (Fig. 3A). Even with the linker regions included (which are variable in length and sequence), an overlay of all five cold-tolerant Ess1s (Fig. 4A) has an overall Cα root mean square deviation (RMSD) that is very low (<0.7), indicating that they likely adopt structures very similar to that of ScEss1.

However, the models revealed several important differences between the *S. cerevisiae* and polar Ess1s. First, the linker that joins the WW and catalytic domains, which is highly structured and contains a prominent α helix in ScEss1 and CaEss1, is longer in the polar Ess1s and appears to be predominantly unstructured, likely providing additional flexibility between the two domains (Figs. 1D and 3A). Second, as observed for other cold-adapted enzymes (*8*, *9*, *43*), the polar Ess1s display a sharp reduction in basic surface charge density and an increased acidic surface charge density (Fig. 3B). In all, about a dozen surface-exposed lysine residues in ScEss1 are changed to shorter, uncharged, acidic, polar, and even hydrophobic residues (e.g., R, G, N, Q, D, S, and L) in the polar enzymes. Changes in surface charge density are thought to contribute to solvation, flexibility, and intracellular diffusion rates in the cold (*11*, *43*, *44*). Third, while the basic architecture of the active site and the key catalytic and substrate binding residues (H64, C120, S122, Q138, F141, and H164) (*25*) are invariant (with the exception of Q138>H in NvEss1) (Fig. 4, A and B), there are several potentially destabilizing substitutions surrounding the active site that could enhance substrate binding and/or catalytic activity in the cold, three of which are shown (Fig. 4C, i to iii). For example, L63 in ScEss1 is buried deep within the core of ScEss1, likely forming a stabilizing hydrophobic interaction with F110, but in four of five polar Ess1s, it is replaced by a serine, a polar residue (no pun intended) (Fig. 4D, i). Moreover, substitution of lysine residues at key positions (e.g., K87 and K167) would eliminate salt bridges that bolster the β-strand structure that forms the backbone of the active site, thus increasing active-site mobility (Fig. 4E) (see Discussion). Last, interdomain flexibility might be increased by key substitutions, for example, at positions E26 and E40 in ScEss1 that are replaced by prolines in the polar Ess1s, disrupting a salt bridge (E26-R59) and potential hydrogen bond (E40-H50) that likely stabilize the interaction between WW and catalytic domains (fig. S7). However, experimental evidence is needed to demonstrate the importance of these and other substitutions on cold adaptation.

### Unique LLPS properties of polar CTDs

The localized sequence divergence of polar CTDs (positions Y1, T4, and S7) and their compromised activities in *S. cerevisiae* prompted us to investigate their biophysical properties. Like many IDRs in proteins, the CTD in yeast and humans is thought to undergo LLPS or to be sequestered into droplets formed by other proteins (*18*). Here, LLPS will refer to in vitro reactions with purified proteins (without crowding agents) that drive droplet formation by liquid-liquid demixing. In contrast, the term phase separation will be used to include in vivo, multicomponent, or less well-characterized biomolecular condensate formation that may occur by various mechanisms (*45*).

We hypothesized that the sequence differences in the polar CTDs would have a notable impact on their ability to undergo LLPS. To begin our analyses, we carried out computational predictions of the degree of intrinsic disorder (*46*) of the polar yeast Rpb1 subunits and their propensity to undergo LLPS (*47*). All the CTDs are predicted to be intrinsically disordered and to undergo LLPS (fig. S8). Interestingly, the CTDs with the highest predicted propensities to undergo LLPS (*A. pullulans* and *H. werneckii*), like ScCTD, complemented better at 18°C (Table 1, left) than those with slightly lower propensities to undergo LLPS (*W. ichthyophaga*, *D. cryoxerica*, and *N. vishniacii*).

**Fig. 5.**
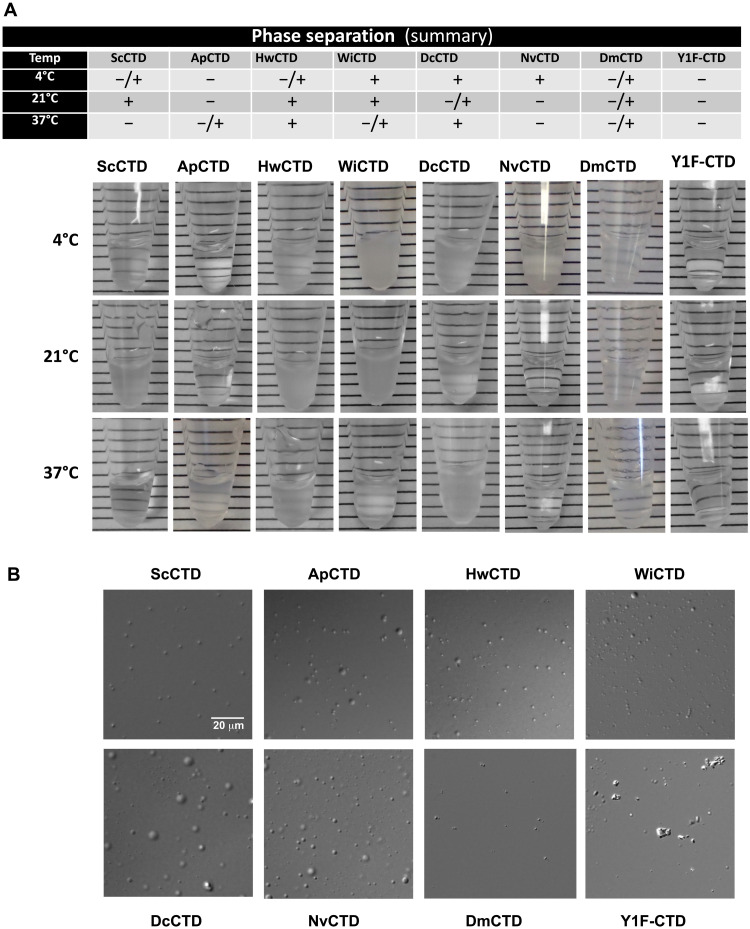
Polar yeast CTDs each undergo LLPS under different conditions. (**A**) Photos of microfuge tubes containing ~100 μl of the indicated SUMO-CTD fusion protein. Highly concentrated (200 to 400 μΜ) purified proteins were dialyzed into a low-salt buffer [220 mM KCl and 20 mM Hepes (pH 7.4)] at low temperature (~4°C) and then screened for LLPS at increasing supplemental salt concentrations (from 0 to 2.0 M additional NaCl added) and at various incubation temperatures. The 2.0 M NaCl samples at the indicated temperatures are shown. *N.b.* no crowding agents were added. Additional data are summarized in table S2. (**B**) Micrographs of droplets formed without added salt or crowding agents, visualized at ×40. The scale for each micrograph is the same.

To experimentally demonstrate LLPS, we fused the polar CTDs to a carrier protein (SUMO) and purified the fusion proteins from *Escherichia coli*. This technique allows high-level protein expression and purification, and is a good mimic for endogenous Rpb1, which has an N-terminal globular domain and a C-terminal intrinsically disordered CTD. Initial experiments were done with highly concentrated proteins (200 to 400 μM) and screened for LLPS (i.e., cloudiness) at increasing salt concentrations (up to 2.0 M NaCl) and various incubation temperatures (table S2). No crowding reagents were included as in a previous study (*18*), where phase separation occurred at lower CTD protein concentrations (20 μM) but required the presence of 16% dextran. Even this relatively crude visual assay revealed stunning differences in behavior among the different CTDs (Fig. 5A). At different temperatures, different CTDs ranged from being completely clear to being partially or fully cloudy, often in a nonlinear (nonpredictable) way. To confirm that the observed cloudiness was from LLPS and not aggregation due to the high protein concentration, we performed differential interference contrast microscopy (Fig. 5B). Proteins that undergo LLPS form droplets that are dynamic in nature, docking and fusing (like oil droplets in water) depending on their surface tension and visco-elastomeric properties (*48*). All the polar CTDs and the *S. cerevisiae* CTD control (but not a Y1F mutant; see below) formed droplets, with some showing uniform-sized droplets [ScCTD, HwCTD, WiCTD (*W. ichthyophaga* CTD), and DmCTD (*D. melanogaster* CTD)] and others a range of sizes [e.g., ApCTD, DcCTD (*D. cryoxerica* CTD), and NvCTD (*N. vishniacii* CTD)]. The significance of these differences is not yet known.

**Fig. 6.**
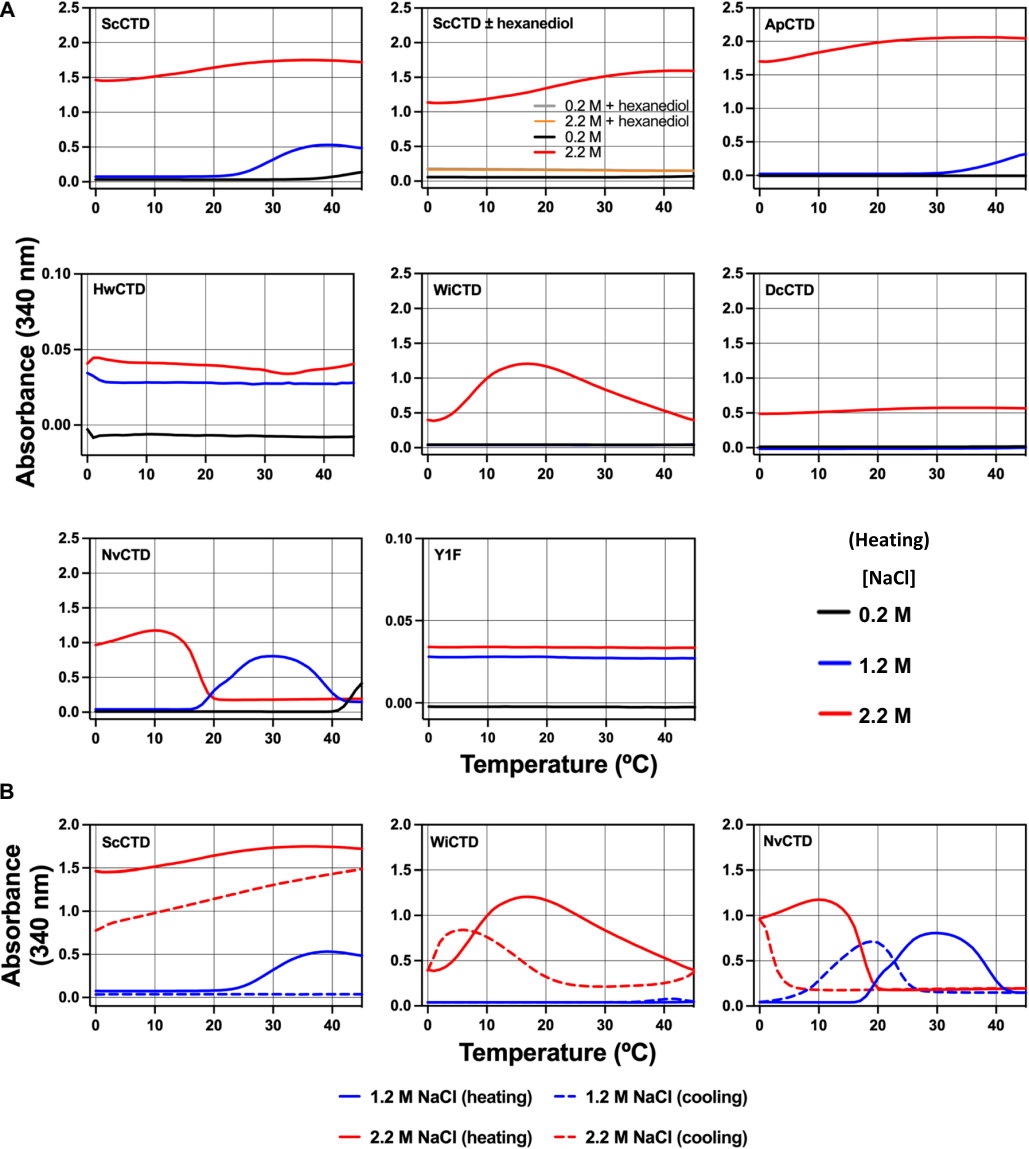
Light scattering assays reveal distinct LLPS profiles for different CTDs. (**A**) Each panel represents scattering profiles of the indicated SUMO-CTD fusion protein (100 μM) at three different salt concentrations: ~ 0.2, 1.2, and 2.2 M (220 mM KCl + 0, 1.0 M and 2.0 M NaCl, respectively). Light scattering was continuously monitored at 340 nm, as the purified proteins were heated from ~0° to >40°C. Note that some (ScCTD, ApCTD, and, to a limited extent, DcCTD) undergo a standard LCST phase separation, while others (NvCTD and WiCTD) undergo dual LCST/UCST phase separation (see text). HwCTD and Y1F ScCTD did not show any LLPS under these conditions. (**B**) Selected LLPS profiles of CTDs in which heating curves (solid lines) and cooling curves from 45° to 0°C (dashed lines) are superimposed. The nonequivalence indicates a hysteresis effect (see text).

To systematically analyze the ability of the CTDs to undergo LLPS, we used a sensitive light scattering assay (*49*) under controlled conditions. We chose two concentrations of protein (50 and 100 μM SUMO-CTD in a low-salt buffer) supplemented with additional concentrations of salt (0.5, 1.0, or 2.0 M NaCl). LLPS was monitored at 340 nm as a function of temperature (0° to 50°C heating or 50° to 5°C cooling). Both 50 and 100 μM CTD-containing solutions showed evidence of phase separation, as measured by distinct changes in light scattering (Fig. 6 and fig. S9). Most notable is that the light scattering profiles for the CTDs from each species are unique. This mirrors the results of the visual assays (Fig. 5) and reveals an interesting biphasic LLPS behavior for some of the CTDs (*W. ichthyophaga* and *N. vishniacii*): As the temperature increases, the amount of phase separation first increases and then decreases. This behavior is defined by a dual phase separation property endowed within these CTDs (see below). The light scattering profiles in all cases show temperature, salt, and CTD concentration dependence, as expected for LLPS. For example, at higher salt, the temperature required for the NvCTD and DcCTD to undergo LLPS is lowered (Fig. 6). Further demonstrating that the observed effects are LLPS, addition of 1,6-hexanediol (*18*) dissolved the ScCTD condensates as indicated by a loss of light scattering (Fig. 6, top row).

To test whether the CTD LLPS process was reversible, we cooled the heated samples and compared the phase transition profiles. In all cases, the process was reversible, but the heating and the cooling profiles were not identical (Fig. 6B and figs. S9 and S10), indicating a hysteresis or kinetic effect. These results are consistent with theoretical behaviors described by polymer physics (*48*).

Although our experiments used high-salt conditions, note that, in contrast to many published studies on CTD LLPS, no crowding agents (e.g., dextran, Ficoll, or polyethylene glycol) or binding partners (e.g., RNA and other proteins) were used in any of our assays. This confirms that LLPS was driven by protein and solution conditions alone and is not the result of co-phase separation of the CTDs into condensates nucleated by crowding agents or other macromolecules. In summary, our data demonstrate that divergent CTDs from different species undergo LLPS in vitro with distinct characteristics that may explain the differences in their abilities to function in a given host organism in vivo. It is tempting to speculate that these unusual behaviors also contribute to temperature and/or salt tolerance of RNAPII in their respective organisms.

### Regulation of CTD-LLPS by temperature

The effect of temperature is one of the most studied factors that regulate LLPS. Temperature-induced LLPS is characterized by two main mechanisms: lower critical solution temperature (LCST), below which the solution is miscible (stays mixed), but above which phase separation will occur, and upper critical solution temperature (UCST), above which the solution stays miscible, but below which phase separation will occur (*49*, *50*). We found that ScCTD, DcCTD, and ApCTD underwent a typical LCST transition, albeit at different temperatures, displaying increased light scattering (cloudiness) with increasing temperature, which, as expected, is enhanced by higher protein and salt concentrations (Fig. 6). In contrast, HwCTD, which underwent LLPS at the high concentrations (200 to 400 μM) used in our crude assay, did not show measurable phase transition at 50 or 100 μM used in our light scattering assays.

NvCTD and WiCTD did not undergo simple LCST or UCST transitions as a function of increasing temperature (Fig. 6). While most proteins (and other polymers) that undergo LLPS favor either LCST or UCST, some display both propensities (*50*). If a protein undergoes both LCST and UCST transitions, *T*_LCST_ < *T*_UCST_, that protein will turn cloudy only at temperatures between *T*_LCST_ and *T*_UCST_, but will remain clear at temperatures below *T*_LCST_ and above *T*_UCST_. Such phase transition behavior is known as the “closed loop” profile. This is exactly what we observed for both NvCTD at intermediate salt concentrations (Fig. 6, blue line) and WiCTD at high salt concentrations (Fig. 6, red line), where each underwent an LCST plus UCST closed loop profile (where *T*_LCST_ < *T*_UCST_). These CTDs add to the few examples of naturally occurring protein sequences that shows such dual behavior (*51*).

### Polar CTDs show aberrant localization and phosphorylation in *S. cerevisiae* cells

The CTD is implicated in the formation of dense RNAPII foci within nuclei of actively transcribing cells. These foci are thought to be composed of biomolecular condensates whose formation is driven by phase separation and regulated by phosphorylation (*18*, *21*, *52*). The foci are most easily visible in mammalian cells; likely contain high densities of RNAPII, transcription factors, co-regulators, target gene enhancers, and promoters; and are thought to be required for efficient gene transcription [reviewed in (*52*)]. On the basis of our data, we hypothesized that polar CTDs, when expressed in *S. cerevisiae*, would show differences (defects) in nuclear focus formation because of their distinct phase separation properties. Such differences might explain their reduced ability to support growth in *S. cerevisiae* under certain conditions.

**Fig. 7.**
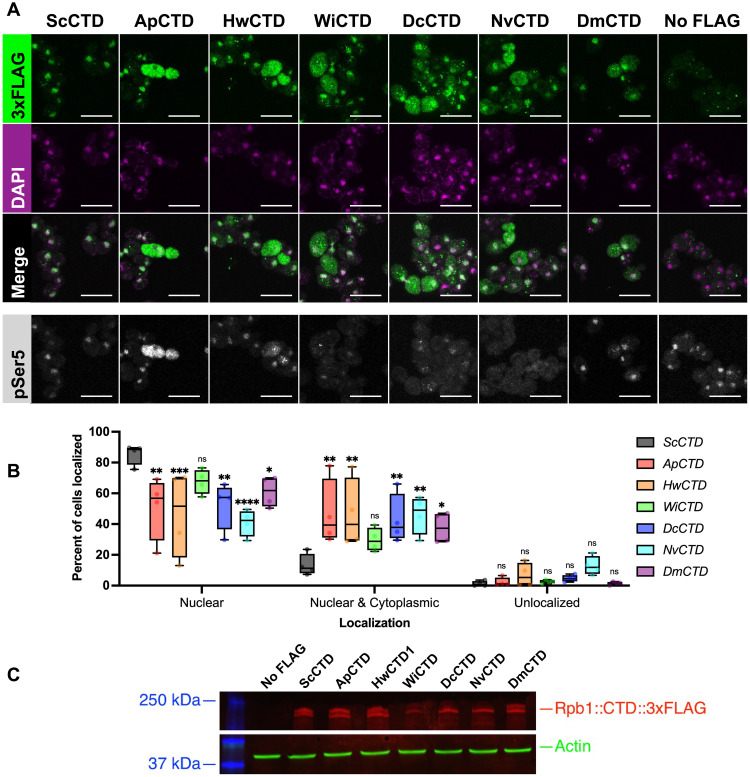
Polar CTDs show defects in Rpb1 nuclear localization. Immunofluorescent staining of fixed *S. cerevisiae* cells expressing the indicated polar yeast CTD. (**A**) Cells grown at 30°C in CSM-His liquid medium were fixed and double-immunostained with rabbit anti-FLAG to detect the C-terminal FLAG-tagged Rpb1-CTD_n_ fusions and rat monoclonal anti-pSer5-CTD antibodies (3E8) to determine the levels of Ser5 phosphorylation within the CTD. Nuclei are identified by 4′,6-diamidino-2-phenylindole (DAPI) staining. Scale bars, 10 μm. (**B**) Subcellular localization of Rpb1::CTD::3×FLAG was scored as being localized to the (i) nucleus, (ii) nucleus and cytoplasm, or (iii) unlocalized (dispersed with no distinct nuclear accumulation). Experiments were performed in biological quadruplicate. None of the cold-tolerant CTDs directed Rpb1 exclusively to the nucleus in *S. cerevisiae* cells as does the “wild-type” ScCTD under these conditions (or at 18°C; figs. S11 and S12). Data are represented as interleaved box-and-whisker plots. *P < 0.05, **P < 0.01, ***P < 0.001, and ****P < 0.0001. ns, not significant. *n* = 688 (ScCTD), 576 (ApCTD), 429 (HwCTD), 624 (WiCTD), 516 (DcCTD), 442 (NvCTD), and 429 (DmCTD). (**C**) Western analysis to monitor expression levels of the polar yeast Rpb1-CTD constructs. Cells were grown at 30°C, and fractionated extracts were reacted with anti-FLAG plus anti-actin antibodies. The No FLAG lane contains protein extract from cells expressing untagged ScCTD as a negative control. DmCTD is a positive control expressing a *D. melanogaster* CTD fusion. Differences in localization in (A) are not explained by modest differences in protein levels detected by Western blot.

To test this hypothesis, we compared localization of Rpb1 in yeast cells bearing different FLAG-tagged CTDs using immunofluorescence staining (Fig. 7, A and B). Control cells expressing the wild-type ScCTD showed almost exclusively nuclear staining, with some areas of highly concentrated nuclear signal (puncta). Budding yeast cells are small (~5 μm in diameter) relative to mammalian cells (https://bionumbers.hms.harvard.edu/KeyNumbers.aspx), and thus, discrete nuclear foci (transcription factories) are difficult to visualize. Nonetheless, staining of the wild type was predominantly nuclear and showed a regular pattern. In contrast, the polar CTDs showed lower levels of nuclear staining, greater cytoplasmic staining, unlocalized puncta that are just as likely to be in the cytoplasm as the nucleus, and a generally irregular pattern of staining (as well as different-sized cells). These differences were also evident in cells grown at 18°C (figs. S11 and S12A).

These experiments were done with cells fixed rapidly in situ, so we do not think that degradation of the polar Rpb1-CTDs was responsible for staining differences. Moreover, Western blot analysis at 30°C (Fig. 7C) and 18°C (fig. S11C) rules out the simple idea that staining differences are caused by differences in polar CTD expression. Instead, we propose that CTD sequence divergence, which is responsible for differences in LLPS in vitro, affects the localization of Rpb1, consistent with results using a *Drosophila* model (see Discussion) (*53*). However, we cannot be certain that the observed localization differences are directly due to changes in phase separation, nor whether they are responsible for the failure of the polar CTDs to fully complement in *S. cerevisiae*. Establishing a causal relationship will require high-resolution and other biophysical approaches.

Sequence divergence and altered phase separation could also affect (and be affected by) posttranslational modification of Ser2 and Ser5 within the CTD, contributing to the differences in their ability to complement in *S. cerevisiae*. For example, different compartmentation via phase separation could alter the access to CTD kinases and/or CTD phosphatases. To address this, we stained cells using monoclonal antibodies that specifically recognize phosphorylation of Ser2 or Ser5. We observed a sharp reduction in levels of phospho-phospho-Ser5, the major target of Ess1, particularly in the CTDs of the halophile (WiCTD) and in both Antarctic species (DcCTD and NvCTD) (Fig. 7A and fig. S11A), precisely those that were least able to complement at 18°C (Table 1). We also observed strong reductions in phospho-Ser2 levels in all the polar species (fig. S12, A and B). The loss of the upper bands in the anti-FLAG Western blots of WiCTD, DcCTD, and NvCTD (Fig. 7C and fig. S11C) is consistent with reductions in phosphorylation. An important caveat is that the divergent sequences in the polar CTDs may alter recognition by the phospho-specific antibodies, which were generated against consensus CTD sequences. Thus, we cannot be certain whether phosphorylation of Ser2 and Ser5 is reduced in the polar CTDs. This could be addressed using proteomic approaches.

## DISCUSSION

As a first step toward understanding how gene transcription occurs in the cold, we cloned and characterized two key regulators of RNAPII transcription from species that live in geographically distant, extreme cold weather environments. Together, Ess1 and the RNAPII CTD form the axis of a dynamic machine whose conformational flexibility provides the motions necessary for efficient transcriptional cycling and cotranscriptional mRNA processing. We found that polar Ess1s are structurally and functionally conserved, showing robust activity in the mesophilic model host. Cold adaptation of Ess1 orthologs likely involves subtle changes that would increase protein flexibility. By contrast, CTDs from polar RNAPII enzymes are highly divergent and displayed distinct LLPS behaviors in vitro, which we interpret as reflecting the evolutionary selection for life in extreme environments and explains why they were not fully functional in the model host. Our findings lay the groundwork for future studies using a new and highly tractable model for cold adaptation of a globular enzyme (Ess1/Pin1) and its intrinsically disordered substrate, as well as for uncovering the link between evolutionary selection for sequence divergence in IDRs, phase separation properties, environmental adaptation, and the CTD code.

### Localized divergence within CTDs enables function and regulates LLPS

The CTDs from cold-tolerant yeasts, when fused to the globular domain of the Rpb1 subunit, only partially complemented in *S. cerevisiae*, depending on the growth conditions (temperature and chemical stress). In general, the more divergent the CTD (e.g., DcCTD and NvCTD), the less well it could complement. While the length of the polar CTDs was conserved (~24 to 28 heptad repeats versus 26 heptad repeats in ScCTD), there was substantial diversity at positions Y1, T4, and S7 of the heptad repeat (YSPTSPS). However, the Ser-Pro motifs were essentially invariant (Fig. 1C and figs. S1 and S2), suggesting that the requirement for CTD prolyl isomerization and/or proline-dependent phosphorylation is absolute, but other properties (and residues) are evolutionarily malleable and optimized for life in different environments.

Notably, not all species have the same substitutions (Fig. 1C and fig. S1), and these substitutions are not uniform throughout all the CTD repeats in a given organism. The substitutions vary between repeats within an organism, and the frequency and patterns of repeat substitutions vary between organisms (fig. S2). Substitutions at positions 1, 4, and 7 in the CTD that affect phase separation are not random. Only nine different amino acids are represented, and among these, by far, the most prevalent substitutions are Y to F in position 1 and S to A in position 7. Phe is known to participate in π-π and cation-π interactions, and Ala could increase flexibility enabling transient interactions, both of which can influence phase separation (*47*). These substitutions could also affect phosphoregulation.

Previous work in yeast and mammals also show that Y1, T4, and S7 are subject to posttranslational modification, primarily phosphorylation, and that these residues play a variety of roles in elongation, anti-termination, and suppression of cryptic transcription (*13*, *17*). Mutations in T4 and S7 are viable in yeast but are sensitive to stress (*54*), whereas a complete Y1F substitution mutation is lethal (*55*). We found that the complete Y1F-CTD mutant was unable to undergo LLPS under all conditions tested (Figs. 5 and 6 and table S2), indicating the importance of this Tyr residue for LLPS and perhaps explaining its inability to support growth in *S. cerevisiae*. Notably, none of the polar CTDs are completely substituted at this position.

Our results are consistent with genetic results from the Stiller laboratory (*56*, *57*) using *S. cerevisiae* that posit that (i) the minimal functional unit spans two repeats and requires three consecutive S-P motifs (“S_2_P-S_5_P-S_9_P” in their nomenclature) (polar CTDs have uniformly conserved S-P motifs), (ii) two tyrosine residues spaced one interval (seven amino acids) apart are required (polar CTDs all have multiple repeats where this is true), (iii) the overall length and not the exact number of functional (or consensus) repeats is critical for growth (the lengths of the polar CTDs are similar to ScCTD, but not the number of conserved repeats), and (iv) deviations from the native sequence within an organism are likely to compromise growth under nonideal conditions (temperature and chemical sensitivities of our host cells expressing polar CTDs). Moreover, our results showing the relationship between CTD sequence and LLPS are consistent with their finding that structured insertions in the CTD (that would interfere with LLPS) are more deleterious in vivo than nonstructured insertions.

**Fig. 8.**
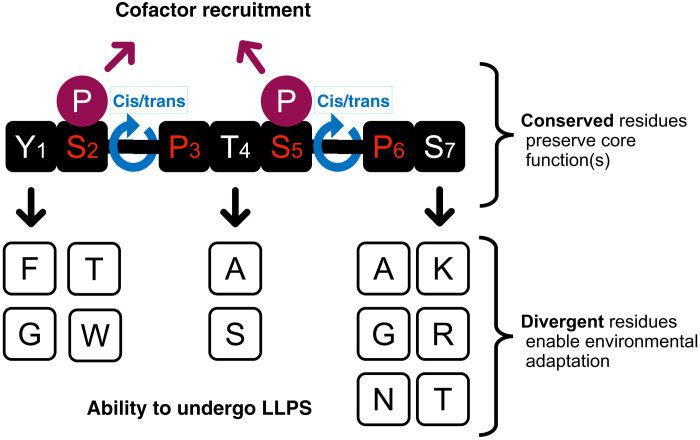
Distinct evolutionary pressures lead to differential conservation within the heptad repeat of the CTD. The CTD is composed of a consensus heptad repeat (YSPTSPS), but not all residues within this motif are conserved to the same degree. The S-P motifs, which are targets for modification by CTD kinases, CTD phosphatases, and the Ess1 prolyl isomerase, are almost universally conserved. Phosphorylation of S2 and S5 and the cis/trans isomerization of the Ser-Pro peptide bonds affect the conformational flexibility of the CTD and are key to recognition and binding by cofactors that are recruited to RNAPII to assist in multiple stages of transcription and RNA processing. In contrast, residues at positions 1, 4, and 7 are variable, but their mutations are nonrandom: The most common substitutions are indicated under the consensus sequence. While Y1, T4, and S7 can be modified by phosphorylation, most residues substituted at these positions are not subject to this modification. We propose (see text) that variability at positions 1, 4, and 7 is an adaptation facilitating survival at different environmental conditions, which directly affect the biophysical properties of the protein (e.g., the ability to undergo LLPS) or trigger a CTD-driven fine-tuning of gene expression. The length of the CTD (i.e., number of heptad repeats) appears to be well conserved within certain phylogenies, e.g., fungi having ~26 repeats and mammals having ~52 repeats.

How would sequence divergence in the CTD facilitate cold- and salt-tolerant growth? First, amino acid substitutions might enhance recruitment of protein cofactors required for transcription, but this would require coevolution of multiple cognate proteins to accommodate those changes in the CTD. While this is possible, we instead favor the idea of a more generalized effect of these substitutions that favor growth at low temperatures, such as a change in the overall biophysical properties of the CTD, consistent with our LLPS data. The CTDs from cold-tolerant yeasts showed marked differences in their ability to undergo LLPS at different temperatures and salt concentrations and to drive nuclear localization of Rpb1-CTD-FLAG within cells. Coupled with their varying abilities to complement in a *S. cerevisiae* host, we propose the following model (Fig. 8). The CTD heptad repeat is sufficiently conserved to retain basic functions in protein recruitment and CTD code regulation such as Ser2 and Ser5 phosphorylation and Ser2-Pro3 and Ser5-Pro6 isomerization, thus fulfilling its critical role in the transcription cycle. But superimposed on this is the evolutionary pressure to promote condensation of the transcription machinery (i.e., formation of transcription factories) enabling function even under extreme conditions (*20*). The phase separation properties of individual CTDs might also be influenced by intracellular interactions with other macromolecules and the production of compatible solutes such as glycerol and trehalose common in cold-adapted yeasts, which would likely act as crowding agents. Thus, the extant sequences are the result of two opposing selective pressures (i) to maintain a consensus-like heptad sequences (and flexibility) for efficient protein recruitment and (ii) to evolve divergent heptad sequences to enable phase separation tailored to the specific cell and external environment.

### CTD sequence divergence is the rule

Genetic, biochemical, and biophysical studies have historically focused on CTD sequences from model organisms such as *S. cerevisiae*, *C. albicans*, *S. pombe*, *Arabidopsis thaliana*, zebrafish, and human, which all have CTDs in which the consensus heptad (YSPTSPS) dominates (*17*). In contrast, fungal CTDs from species other than those belonging to *Saccharomycetes* show substantial divergence (fig. S2) (*35*). The heptad in most fungal groups thus appears to have evolved under different selective pressures than most model organisms and their relatives. Thus, while the divergent CTDs characterized here have unique LLPS properties that might contribute to cold tolerance, this is probably not unique to polar or halophilic fungi. Many of the changes we observed, such as the Y1F substitutions found in some of the heptad repeats in DcCTD and NvCTD, are also frequent in other species from various taxonomic groups, including *Agaricomycetes*, *Sordariomycetes*, and *Eurotyomycetes*. Thus, phylogenetic analysis (e.g., fig. S2) supports the idea that the Ess1 binding sites/Ser-Pro motifs within the CTD must be retained for function, but that other residues can evolve to suit the local conditions and are inherited in related subgroups.

Using a *Drosophila* model system, the Gilmour laboratory found that replacing all the normally divergent CTD repeats (variation mostly at positions 4 and 7) with consensus heptads was lethal in flies and led to aberrant CTD localization in cells (*53*). When the length (*39*–*42*) was shortened to yeast-like lengths (20 to 29 repeats) or the divergence was retained, CTDs localized correctly and were functional. Their data are fully consistent with our proposal that sequence divergence (and length, according to their work) can dictate phase separation properties, which, in turn, directs localization and function in vivo, and that protein recruitment is not the major driving force for diversification of CTD sequences.

### Phase separation as a versatile mechanism for stress response and adaptation to extreme environments

Recent evidence demonstrates that formation of biomolecular condensates facilitates specific biological processes and provides spatiotemporal control over normal cellular functions by generating favorable microenvironments (*19*). However, phase separation also appears to be used as a mechanism for cellular sensing and adaption to the surrounding environment. For example, in different fungal species, RNA binding proteins Whi3 and Sts5 promote condensate formation in response to heat and nutritional stress, a process regulated by phosphorylation-dependent mechanisms (*58*). Other examples include polyadenylate-binding protein 1 (Pab1), which uses temperature-dependent phase separation as an adaptive response to heat shock (*59*), and Pub1, which forms condensates and acts as a pH sensor following glucose starvation (*60*). Thus, it is reasonable to speculate that organisms living in extreme environments, such as the Arctic and Antarctic, will evolve cellular mechanisms that use phase separation to respond to temperature extremes and enable growth in harsh conditions.

### Is there a CTD-LLPS code?

Experimental and computation analysis indicates that different amino acid compositions and the linear sequence dictate distinct aspects of the LLPS process (*61*). Some residues drive LLPS, whereas others dictate the material properties of the condensates or the propensity to undergo UCST versus LCST (*62*). The strength and time scale of phase separations are determined by sequence-specific interactions that include electrostatic, π-π, cation-π, and hydrophobic interactions, as well as valency and patterning of the amino acids within the protein sequence (*63*). Extrinsic conditions, such as ionic strength, temperature, and macromolecular crowding, also modulate LLPS properties. Deciphering the “rules” for LLPS is currently a major challenge in the field. For example, in elastomeric proteins, resilins and tropoelastins are both enriched with Gly- and Pro-containing repeats, but resilins undergo UCST, while tropoelastins undergo LCST. Other residue identities likely account for the difference; resilins are enriched in charged (with Arg>Lys) and polar residues, while tropoelastins are enriched in nonpolar hydrophobic residues and depleted in negative charges, but with a preference of Lys>Arg positive charges (*62*). We note that the relatively simple repeat sequence found in the RNAPII-CTD, as well as the naturally occurring divergence among species, provides a wealth of opportunity to dissect the contributions of length, composition, and sequence of individual amino acids within the CTD to help elucidate an “LLPS code.” In addition, it is possible or even likely that phosphorylation and prolyl isomerization of the S-P motifs within the CTD also contribute to propensity to undergo phase separation, providing another avenue for regulation.

### Polar Ess1 orthologs show changes associated with cold adaptation

While the Ess1 enzymes from cold-tolerant fungi are highly conserved in function and overall structure, they nonetheless exhibit changes that reflect adaptation to life in the cold. Enzymes from cold-adapted organisms use a variety of mechanisms to retain catalytic activity at low temperature (*7*–*9*, *43*, *64*). Chief among these is to maintain flexibility in and around the active site. The adaptations can be global, such as an overall decrease in internal hydrophobicity of the protein core and/or increased surface hydrophobicity, and fewer stabilizing salt bridges or hydrogen bonds, or they can be subtle, such as one or a few key amino acid substitutions governing the motions in reaction core. While these changes can lower substrate binding affinity (higher *K*_m_), they also lower the necessary free energy change required to reach the transition state intermediate for catalysis, effectively raising the reaction rate (higher *k*_cat_) at low temperatures (*8*). While we have not measured the catalytic activity of the cold-tolerant Ess1s, nor conducted thermal denaturation studies, we have identified changes in the structural models that predict adaptations to increase flexibility or decrease barriers to substrate binding.

The polar Ess1 models are similar in overall structure, with conserved globular domains (WW and catalytic) connected by a variable linker region. However, the linker region, which in ScEss1 is relatively short (K46-R59), is highly ordered (*25*), and contains a three-turn α helix, is longer (12 to 25 residues) in the polar Ess1s, is rich in residues prone to flexibility (G, Q, and E) (*65*), and has prolines that would preclude long α helices (Figs. 1D and 3A). These unstructured linkers and the previously described proline substitutions at positions E26 and E40 in ScEss1 (fig. S7) likely add to interdomain flexibility and/or mobility within the WW domain. Given that the WW and catalytic domains cooperate to simultaneously bind long, multivalent CTD substrates (*25*), the added linker length and interdomain flexibility in the polar Ess1s might compensate for substrates that would be “stiffer” in cold conditions.

The altered surface charge distributions in the polar Ess1s were pronounced (Fig. 3B) and were similar to those reported for other cold- and salt-adapted enzymes (*66*). Current models suggest that reducing total surface charge, mostly by substituting basic surface-exposed lysine residues, and increasing surface hydrophobicity help maintain flexibility (*8*, *43*). In addition, changing surface charge from highly basic toward acidic can increase intracellular protein diffusion rates (*67*), which, for the cold-tolerant Ess1s, would facilitate its movement within cells. Accordingly, increased negative charge is associated with activity in high-salt environments, which are often linked to cold habitats (*43*, *66*, *68*). We identified a dozen substitutions of surface-exposed Lys residues (Fig. 3B) in the polar Ess1 enzymes, several of which were changed to hydrophobic or acidic residues.

A few of these lysine substitutions are likely to have measurable effects beyond solvent interactions. K167 in ScEss1 forms a salt bridge with D103, supporting the active-site β-sheet structure on which the catalytic residues are displayed (Fig. 4, C and E, ii). This lysine is replaced by an acidic residue (E) in ApEss1 and HwEss1, or a hydrophobic residue (L) in DcEss1, NvEss1, and WiEss1, neither of which could participate in this stabilizing interaction. The corresponding acidic residue (D103) is also missing or substituted by residues that cannot form a salt bridge. In the Antarctic enzyme DcEss1, another salt bridge, K87-E158, on the opposite side of the active-site scaffold is disrupted, with K87 replaced by a proline (Fig. 4, C and E, iii). Again, this could provide flexibility for engaging and isomerizing substrates in the cold.

The polar Ess1 enzymes also have an increased acidic surface density (Fig. 3B). Negatively charged side chains of Asp and Glu residues in cold- and salt-adapted enzymes engage in electrostatic interactions to order the surrounding water molecules (via hydrogen bonding or dipole interactions), creating a hydration shell that helps keep the proteins solvated and maintain flexibility (*44*). Such a mechanism is used to counter the increased water viscosity due to low-temperature and high-salt environments in which polar microbial life thrives. Whether the numerous additional glutamic and some aspartic acids in the polar Ess1 enzymes help solvate and maintain flexibility remains to be determined.

There are also substitutions in the hydrophobic core that could disrupt packing and further enhance flexibility of the active site. The best example is L63 of ScEss1, which is changed to serine in four of five polar Ess1s (Fig. 4, C and D, i). This disrupts hydrophobic contacts between L63 and F110, which is retained as a hydrophobic residue in all the polar yeast species (F or L). In addition, this change introduces a polar molecule into the hydrophobic core of the protein, further destabilizing it. Last, this position is located directly behind the catalytic residue H64, allowing more freedom of movement for the β sheet at the center of the active site. Thus, a single substitution may have multiple destabilizing effects on the catalytic domain and the active site.

In summary, our results highlight the multiple evolutionarily pressures on proteins to adapt to extreme environments. The first, which has been well studied, although still not fully understood, is for globular protein domains to retain stability and/or flexibility demanded by temperature and solvent extremes. The second is for intrinsically disordered proteins to adapt their biophysical properties, such as the ability to undergo phase separation, to the environment in which the host organism lives, something we propose here based on data presented for the RNAPII CTD. While previous studies were consistent with this general idea (*35*, *53*, *69*), our results are the most direct demonstration that evolutionary divergence in CTD sequence results in measurable differences in the biophysical properties (LLPS in vitro) and also affects CTD localization and function in vivo. More generally, we propose that environmentally tuned phase separation could help overcome barriers to enzyme function in other extremophilic organisms.

## MATERIALS AND METHODS

### Fungal isolates and genomic information

Whole-genome sequencing of *A. pullulans* (isolate: EXF-3645) (*36*), *H. werneckii* (isolate: EXF-2000) (*37*), and *W. ichthyophaga* (isolate: EXF-994) (*32*) was carried out previously, and sequences were deposited at DNA Data Bank of Japan/European Nucleotide Archive (DDBJ/ENA)/GenBank under accession numbers QZBR00000000, MUNK00000000, and APLC00000000, respectively. The genomic sequences for *D. cryoxerica* (isolate type: ANT-03-071^T^ 5CBS 10919^T^ 5PYCC 5967^T^) (*33*) and *N. vishniacii* (isolate type: ANT03–052; CBS 10616) (*34*) were generated by the Joint Genome Institute (JGI). This Whole Genome Shotgun project for *N. vishniacii* has been deposited at DDBJ/ENA/GenBank under accession number JABEVT000000000 and for *D. cryoxerica* at JGI (project ID 1001558).

### Cloning and expression of fungal Ess1 and CTD orthologs

*ESS1*-related sequences were cloned from *A. pullulans*, *H. werneckii*, and *W. ichthyophaga* from cDNA preparations from each species using a two-step polymerase chain reaction (PCR) amplification strategy (PCR conditions for these and other reactions are provided in table S3). The first step used 20– to 30–nucleotide (nt) gene-specific primers (oligonucleotides used in this study are listed in table S4) and Phusion High-Fidelity Master Mix with GC buffer from New England Biolabs (NEB). In the second step, the initial products were used as templates for PCR using primers (35 to 55 nt) with 15-nt 5′-overhangs designed for cloning into plasmids using HiFi Assembly (NEB). The PCR products were recombined into the Eco RI–digested Hanes laboratory expression vector pJGS4-4_0_, an *amp^R^*, *TRP1*, 2 μM yeast–*E. coli* shuttle vector derivative of pJG4-1ΔE using NEBuilder HiFi Assembly (NEB). An N-terminal hemagglutinin (HA) tag was added by including a single-stranded oligo in the three-piece assembly reaction. The resulting constructs express HA-tagged Ess1 open reading frames from the strong *ADH1* promoter, followed by stop codons in all three frames and an *ADH1* transcription terminator (fig. S4A).

For the Antarctic species, *D. cryoxerica and N. vishniacii,* the *ESS1* genes were PCR-amplified directly from genomic DNA, which was purified using the Quick DNA Fungal/Bacterial Miniprep Kit (Zymo Research, D6005) from cells grown on yeast extract peptone dextrose (YEPD) plates at 4°C. The products were generated in two steps and cloned into pJGS4-4 as above. *N. vishniacii* sequence analysis revealed a potential short, in-frame intron in the linker region of Ess1, flanked by “GU...AG” splice sites (encoding amino acids 61 to 69; GATEEEEEE). We therefore made two constructs: with and without the potential intron. First, we cloned the version retaining all the encoded sequences into pJGS4-4, and using a variation of the QuikChange method (Agilent), a single oligonucleotide with homology on either side of the intron was used to prime synthesis of the entire plasmid, thereby removing the intron. Both versions of *ESS1* were functional (Table 2). To clone the control *D. melanogaster dodo*, total RNA was prepared from wild-type *Drosophila* ovaries (Bloomington Drosophila Stock Center, #5905) using the Monarch Total RNA Miniprep Kit (NEB) according to the manufacturer’s instructions. One hundred nanograms of total RNA was reverse-transcribed with ProtoScript II (NEB). cDNA was amplified and cloned via HiFi Assembly in pJGS4-4 as above.

To clone the CTDs of the RNAPII’s largest subunit from cold/salt-tolerant fungi and express them as SUMO fusions *in E. coli*, CTD-related sequences from *RPO21* (which encodes Rpb1) were amplified from cDNA or from genomic DNA as described above. PCR products were recombined, using HiFi Assembly, into a linear (PCR-generated) pET-SUMO vector. The resulting plasmids express 6×-His-SUMO-CTD with thrombin and SUMO protease sites at the 6×-His-SUMO and SUMO-CTDs junctions, respectively (fig. S4A). Two variants of the CTD from *H. werneckii* (Hw1 and Hw2) and *D. cryoxerica* (Dc1 and Dc2) were cloned. Hw2 (GenBank, OTA33161.1) has a 14–amino acid deletion (SPAYQVTSPRFSPA) and two other single amino acid substitutions (S>A and A>G), relative to Hw1 (GenBank, OTA34258.1). Dc1 (DC_264154) and Dc2 (DC_330815) have several polymorphisms throughout their lengths. In *H. werneckii*, the two isoforms belong to separate genome copies that were merged to form a diploid hybrid strain used in this study (*38*). It is not certain whether these variant CTDs are due to gene duplication (paralogs) of the Rpb1 gene within otherwise haploid fungi, or hetero-alleles within a diploid.

To express *S. cerevisiae* Rpb1 subunits bearing the CTDs from the cold/salt-tolerant fungi, we used plasmid pFR467, a derivative of pRS313 (*CEN HIS3*) that expresses a CTD-less Rpb1-3×FLAG under the control of the *RPB1* promoter and terminator (*55*). Fungal CTDs were amplified from pET-SUMO-CTD clones (see above) and recombined into Mlu I–digested pFR467 by HiFi Assembly. This set of constructs contained a stop codon immediately following the CTDs to express untagged proteins. A second set of constructs was made (with the first set as PCR templates) using modified downstream primers to remove the stop codon, enabling read through into a 3×FLAG-epitope tag for detection with anti-FLAG antibodies (see fig. S4A).

### Phylogenetic analyses

Amino acid sequences of Ess1 and Rpo21 proteins were aligned with Mafft 7.745 (*70*). Rpo21 alignment was then separated into the CTD region, which was analyzed separately as described below, and the core region, which was used for phylogenetic analysis. The estimation of phylogeny was performed in IQ-TREE 2.0.3 (*71*) by first estimating the best amino acid substitution model, which used the matrix estimation (LG) for both alignments. Evolutionary rate heterogeneity was estimated as gamma distribution with four categories and alpha shape parameter 1.2346 or 0.9688, and with 0.1176 or 0.1428 invariable sites for Ess1 and Rpo21, respectively. Branch supports were calculated as SH-aLRT values. The CTD region of Rpo21 was analyzed with MerCat 0.2 (*72*) to determine the main amino acid 7-mers and then manually to identify the characteristic YSPTSPS (and related) heptamer repeats, and the repeats were visualized using Inkscape software 1.0.2. (https://inkscape.org/).

### *S. cerevisiae* strains, media, curing, and growth assays

Yeast strains were cultured using standard methods and media, YEPD, and complete synthetic medium (CSM) with relevant amino acid dropouts, as described by Sherman (*73*).

To test cold/salt-tolerant fungal Ess1 function, complementation assays (curing experiments) were performed using *S. cerevisiae* strain YPR-57 [*MAT***a** *ade2-1 can1-100 his3-11,15 leu2-3,112/LEU2 ura3-1 ess1*Δ*::HIS3,* pGD-CaESS1 (*CaESS1*, 2 μM, *URA3*)] (*74*). This strain contains a chromosomal deletion of the essential *ESS1* gene, covered by a *URA3*-marked plasmid expressing a genomic CaEss1 fragment that is known to complement (*12*). This strain enables plasmid shuffling [carried out essentially as described (*74*)]. Here, cells were transformed with the respective polar *ESS1* plasmid (*CEN TRP1*), and 5-ml cultures of individual transformants were grown in CSM-Trp medium for five successive overnights (1:25 dilutions each day) at 18°C and plated onto CSM-Trp plates. Single colonies were picked onto grids and scored by replica plating onto the appropriate selective medium (CSM-Ura, CSM-His, and CSM-Trp). All plating and scoring were done at 18°C. The % curing was calculated among His^+^Trp^+^ cells as number of Ura^−^ colonies/total × 100. To test for CTD function, we used *S. cerevisiae* strain CBW1 [*MAT***a** *ura3-1 trp1-1 leu2-3,112 can1-100 ade2-1 his3-11,15 rpb1*Δ::*kanMX*, pRP112 (*RPB1*, 2 μM, *URA3*)]. Cells were transformed with the respective Rpb1-CTD plasmid (CEN, *HIS3*), and individual transformants were grown at 18° or 30°C for five successive overnights in CSM-His medium. Cells were scored as above using the appropriate selective medium. For curing done at 18°C, constructs expressing the CTDs with no FLAG-tag were used. For curing done at 30°C, constructs expressing FLAG-tagged CTDs were used.

To measure growth rates of cells from curing experiments (i.e., dependent upon polar Ess1 or CTD replacements), overnight cultures were grown in YEPD or CSM dropout medium and grown to mid-log phase [optical density at 600 nm (OD_600_) of 0.5 to 1.0]. Cells were diluted to 0.1 OD_600_ units (about 2 × 10^6^ cells/ml), and growth was monitored in a 500-μl 48-well microtiter-well format using Tecan Infinite F200. To monitor cell growth under different stress conditions (temperature and drugs), cells were grown overnight in selective (liquid) media and serial (1:3) dilutions were spotted onto solid media with or without drug.

To test for CTD rearrangements in vivo that may have enabled growth, plasmids expressing Rpb1-[CTD_polar_] proteins were rescued from 1 ml of saturated cultures of cured cells using a Zymoprep Yeast Plasmid Miniprep II kit (Zymo Research) and eluted in 10 μl of 0.5× TE [10 mM tris-HCl pH 8.0, 1.0 mM ethylenediaminetetraacetic acid (EDTA)] . Five microliters was transformed into 50 μl of 5-alpha high-competent *E. coli* (NEB). Transformation reactions were spun down and resuspended in 200 μl of double-distilled H_2_O, and 125 μl was plated onto LB + ampicillin (50 μg/ml) plates. Resistant cells were identified, plasmid mini-preparations were made using the Monarch Plasmid Miniprep Kit (NEB), and CTD-DNA sequence was determined (by GeneWiz) from two minipreps representing each species.

### Protein expression and purification

Large-scale (10-liter) purifications of 6×-His–tagged Ess1 and SUMO-CTD fusion proteins were carried out at Ichor Life Sciences (Lafayette, NY), essentially as described (*25*). Briefly, 10-liter cultures of *E. coli* transformants [BL21(DE3), NEB] were grown to OD_600_ = 0.6 to 0.8, induced with 100 μM isopropyl-β-d-thiogalactopyranoside (IPTG), and grown overnight at 18°C. Cells were lysed by sonication, and proteins were purified with gravity Ni-NTA agarose (Qiagen) affinity chromatography followed by Sephadex S200 fractionation. Final buffer conditions for purified Ess1 proteins (25 to 145 mg/ml; 1.2 to 7.3 mM) were 20 mM tris (pH 8.0), 250 mM NaCl, and 5 mM dithiothreitol and, for the SUMO-CTD fusion proteins (0.4 to 1.9 mg/ml; 11 to 55 μM), 50 mM Na phosphate (pH 7.4), 300 mM NaCl, 1 mM benzamidine, 200 mM arginine-HCl, 5% glycerol, and 5 mM β-mercaptoethanol, plus protease inhibitors. Additional preparations, when needed (e.g., ApCTD and DcCTD), were made from 2-liter cultures.

### Modeling of polar Ess1 structures

All polar Ess1 primary sequences were entered into the Chimera protein structure visualization software and modeled against the ScEss1 crystal structure [Protein Data Bank (PDB) ID: 7KKF] using the program MODELLER (*75*). The overall Cα RMSD was <0.7 Å for all the modeled structures. Structure comparisons, including surface charge variations, substitutions that disrupt salt bridges, and changes of interior hydrophobic character, were all done through direct observation of overlaid structures in Chimera (*76*).

### LLPS light scattering assays

Each purified SUMO-CTD fusion protein was concentrated by centrifugation at 4000 rpm at 4°C using a 5-kDa cutoff Amicon concentrator before being extensively dialyzed in a phase separation buffer containing 220 mM KCl and 20 Hepes (pH 7.4). Light scattering assays as a function of CTD protein concentration, salt, and temperature (heating/cooling) were performed using an Agilent Cary 3500 ultraviolet-visible double-beam spectrophotometer at 340 nm.

### Protein extraction and Western blot

Overnight yeast cultures were grown in CSM-His at 30°C, diluted in CSM-His medium, and incubated at either 30° or 18°C to mid-log phase (OD_600_ = 0.4 to 0.9). Cells (2.5 OD_600_ units) were harvested, and cell pellets were flash-frozen in a dry ice–ethanol bath or liquid nitrogen. Cell pellets were thawed on ice, resuspended in 100 μl of sterile water, mixed with 100 μl of 0.2 M NaOH, and incubated at room temperature for 5 min. Extracts were resuspended in 50 μl of sample buffer [360 mM tris-HCl (pH 6.8), 12% SDS, 0.015% bromophenol blue, 18% β-mercaptoethanol, and 60% glycerol] and boiled for 5 min. Cell extracts were centrifuged briefly, and 5 to 10 μl of supernatant were loaded onto 4 to 20% TGX gels (Bio-Rad) and fractionated at 100 V for 10 min and 300 V for 20 min. Western transfer, antibody incubations, and washes were performed using PVDF–low fluorescence (LF) membranes with a Trans-Blot Turbo system as described by the manufacturer (Bio-Rad). Membranes were imaged using the ChemiDoc MP Imaging System with Image Lab Touch software (Bio-Rad). Primary antibodies were used at 1 μg/ml to detect 3×FLAG (rabbit anti-DYKDDDDK; Invitrogen, PA1-984B) and actin (mouse anti–β-actin; Abcam, ab170325). The following secondary antibodies were used at 1 μg/ml and were from Jackson ImmunoResearch: anti-rabbit immunoglobulin G (IgG) (711-165-152) conjugated to Cy3 and donkey anti-mouse IgG conjugated to Alexa Fluor 488 (715-545-150).

### Immunofluorescence and confocal microscopy

Overnight cultures were grown at 30° or 18°C in CSM-His, diluted in CSM-His medium, and incubated at either 30° or 18°C, respectively, to mid-log phase (OD_600_ = 0.6 to 1.5). Cell fixation, harvesting, permeabilization, and antibody incubations were performed according to Wei (*77*) with the following modifications: PBT-NDS [1× phosphate-buffered saline (PBS), 3% bovine serum albumin, 0.3% Triton X-100, and 5% normal donkey serum] was used as the blocking buffer and antibody diluent, and slides were washed after each antibody incubation with 0.3% PT buffer (1× PBS and 0.3% Triton X-100). Rat monoclonal antibodies to pSer2-CTD (3E10) and pSer5-CTD (3E8) (*78*) were purchased from Abcam and used at a final concentration of 0.45 and 0.65 μg/ml, respectively. Slides were mounted in VECTASHIELD PLUS antifade mounting medium with 4′,6-diamidino-2-phenylindole (DAPI) (Vector Labs, H-2000). *Z*-stacks were taken with a Leica SP8 confocal microscope and processed with Fiji (*79*). Figures are maximum intensity projections of *Z*-stacks. Cells were counted using the Cell Counter plugin in Fiji (v2.3.0/1.53q).

### Statistical analysis

All experiments were performed at least in duplicate. For curing experiments, at least two independently derived plasmid clones (sequence confirmed) were used to transform yeast and at least two individual transformants for each clone were used for curing. Numbers of colonies screened (*n*) for select phenotypes are given (Tables 1 and 2). All phase transition experiments were done at least three times. Representative data are shown. For immunofluorescence localization, significance was calculated by ordinary two-way analysis of variance (ANOVA) with Dunnett’s multiple comparisons test with a single pooled variance using GraphPad Prism 9.4.

## Notes

View/request a protocol for this paper from *Bio-protocol*.
