# Supplementary material for: Coevolution of the Ess1-CTD axis in polar fungi suggests a role for phase separation in cold tolerance
Source: Sci Adv. 2022 Sep 7;8(36):eabq3235. doi: 10.1126/sciadv.abq3235 (PMC9451162; doi:10.1126/sciadv.abq3235)
Supplement: Supplementary file 1 — Figs. S1 to S12 Tables S1 to S4 [file sciadv.abq3235_sm.pdf]

Science Advances
AAAS

Supplementary Materials for

Coevolution of the Ess1-CTD axis in polar fungi suggests a role for phase separation in cold tolerance

Ryan J. Palumbo *et al.*

Corresponding author: Alaji Bah, baha@upstate.edu; Steven D. Hanes, haness@upstate.edu

Sci. Adv. **8**, eabq3235 (2022)
DOI: 10.1126/sciadv.abq3235

This PDF file includes:

Figs. S1 to S12
Tables S1 to S4

Sequences of Model Organism and Polar CTDs

S-P (Ess1 binding sites) are highlighted in red
Residues that differ from the consensus heptad (YSPTSPS) are indicated in bold
* note that T-P motifs are also Ess1 substrates

Sc CTD

1. FSPTSPT
2. YSPTSSP A
3. YSPTSPS
4. YSPTSPS
5. YSPTSPS
6. YSPTSPS
7. YSPTSPS
8. YSPTSPS
9. YSPTSPS
10. YSPTSPS
11. YSPTSPS
12. YSPTSPS
13. YSPTSPS
14. YSPTSPS
15. YSPTSPS
16. YSPTSPS
17. YSPTSPA
18. YSPTSPS
19. YSPTSPS
20. YSPTSPS
21. YSPTSPS
22. YSPTSPN
23. YSPTSPS
24. YSPTSPG
25. YSPGSPA
26. YSPKQDEQKHNENE NSR

Human CTD (first half)

1. YSPTSPA
2. YEPRSPGG
3. YTPQSPS
4. YSPTSPS
5. YSPTSPS
6. YSPTSPN
7. YSPTSPS
8. YSPTSPS
9. YSPTSPS
10. YSPTSPS
11. YSPTSPS
12. YSPTSPS
13. YSPTSPS
14. YSPTSPS
15. YSPTSPS
16. YSPTSPS
17. YSPTSPS
18. YSPTSPS
19. YSPTSPS
20. YSPTSPS
21. YSPTSPS
22. YSPTSPN
23. YSPTSPN
24. YTPTSPS
25. YSPTSPS
26. YSPTSPN
27. YTPTSPN
28. YSPTSPS
29. YSPTSPS
30. YSPTSPS

Human CTD (second half)

31. YSPSSPR
32. YTPQSPT
33. YTPSSPS
34. YSPSSPS
35. YSPTSPK
36. YTPTSPS
37. YSPSSPE
38. YTPTSPK
39. YSPTSPK
40. YSPTSPK
41. YSPTSPT
42. YSPTTPK
43. YSPTSPT
44. YSPTSPV
45. YTPTSPK
46. YSPTSPT
47. YSPTSPK
48. YSPTSPT
49. YSPTSPK GST
50. YSPTSPG
51. YSPTSPT
52. YSLTSPA ISPDDSDEEN

Dm CTD

1. YSPTSPN
2. YTASSPG
3. GASPN
4. YSPSSPN
5. YSPTSPL
6. YASPRYA
7. STTPNFN
8. PQSTG
9. YSPSSSG
10. YSPTSPV
11. YSPTVQF Q
12. SSPSFAG SGSNI
13. YSPGNA
14. YSPSSSN
15. YSPNSPS
16. YSPTSPS
17. YSPSSPS
18. YSPTSPC
19. YSPTSPS
20. YSPTSPN
21. YTPVTPS
22. YSPTSPN
23. YSASPQ
24. YSPASPA
25. YSQTGVK
26. YSPTSPT
27. YSPPSPS
28. YDGSPGS PQ
29. YTPGSPQ
30. YSPASPK
31. YSPTSPL
32. YSPSSPQ
33. HSPSNQ
34. YSPTGST
35. YSATSPR
36. YSPNMSI
37. YSPSSTK
38. YSPTSPT
39. YTPTARN
40. YSPTSPM
41. YSPTAPS H
42. YSPTSPA
43. YSPSSPT FEESED

Ap CTD

1. YQGGSFS P
2. YSGGQSP G
3. YAPTSPF SMG
4. TSPSSPG
5. YASPSSPG
6. YSPRSPG AAL
7. GSPGYGM
8. GSPASPA
9. YNPTSPT
10. YSPTSPA YGK
11. GSPTSPS
12. YSPTSPS
13. YSPTSPS
14. YSPTSPS
15. YSPTSPS
16. YSPTSPA HRSG
17. PSPTSPR
18. YSPTSPA
19. YSPTSPA
20. YSPTSPS
21. YNPGGAS
22. HSPTSPS
23. YSPTSPV
24. YSPTSPA QQG
25. YSPTSPQ
26. YTPNSPG QASPK
27. YSPTSPK
28. YSPNSPG Q

Hw1 CTD OTA34258

YDMGSPL AEGG
YAGGPDYAAS
FSPIIDAGQADVGGGFTA
YGGGFGGQSPYTGGMSPG

1. YAPTSPF NAG
2. FSPTSPG YGG
3. YSPTSPA G
4. YSPTSPG F
5. DGATSPA
6. YQVTSPR
7. FSPASPA
8. YTPTSPT
9. YSPTSPA
10. YSGGNK
11. VSPTSPS
12. YSPTSPS
13. YSPTSPS
14. YSPTSPN
15. YSPTSPA MHSGSAP
16. GSATSPK
17. YSPTSPQ
18. YSPTSPA
19. YSPTSPA
20. YSPTSPK
21. YGSSSAG AGSMS
22. TSPTSPG
23. YSPTSPV
24. YSPTSPK YGGGGQGASTS
25. NSPTSPS
26. YSPTSPQ
27. YSPNSPR ED

Wi CTD EOR00077.1 (Salt-loving)

MTPYAHGKNSYEAQIIG
DDTALFSPIAASGSDESSK
EYLGYGQSPLVTGGATSPG

1. YSPSSPT
2. WSPTSPG
3. YVPATSP AVGGA
4. VSPWVPQ GGTSPG
5. YSPSSPM
6. VGISSPS
7. YSPSSPK
8. FSPSSPT
9. YSPASPS
10. YSPTSPR
11. YSPASPA
12. YSPSSPK
13. YSPTSPK
14. YSPTSPQ
15. YSPTSPQ
16. YSPTSPK
17. YSPASPA
18. YSPASPG
19. YSPASPA
20. YSPASPA
21. YSPASPA
22. YSPASPA
23. YSPASPA
24. YSPTSPG GATGVNGETKPSWQT GNTSSGPSWKT

Dc CTD 264154(DC1)

YDNFSPM
WAPNGVVGSAA

1. FSPMQTS
2. QNEEGGNFAYMGYGQSPMHGG ASPGG
3. YSPSSPA G
4. YSPTSP
5. FAITSPA
6. YSPTSPF QGAGAASPWVPRGG
7. YGNTSPA
8. YSPSSPQ
9. YSPTSPQ
10. YSPASPS
11. FSPSSPT
12. YSPASPA YAGATRASP
13. YSPASPA
14. YSPTSPL
15. AGITSPR
16. YSPTSPH
17. YSPASPP
18. FSPTSPV
19. YSPASPA
20. FQATSPR
21. YSPASPQ
22. FSPASPS
23. YSPASPL
24. YSPASPA
25. YSPASPA
26. FSPSSPA FGVQRNG

NGTPGPSQQQNGASG
SGAAAPPAAKNKGWG
NTGYAASPSWKS

Nv CTD

1. YSPTSPS
2. YSPTSPF ITSPAH
3. TSPASPA
4. YGGASPW IGVGIGGPTSPA
5. YSPHSPA
6. YASPASP G
7. FSPSSPM G
8. YSPASPS
9. FSPGQ
10. YSPASPS
11. YSPTSPH GA
12. YSPTSPA
13. FSPASPA
14. FSPASPA
15. FSPASPA
16. FSPASPA
17. FSPASPA
18. FSPASPA
19. FSPASPA
20. FSPASPA
21. FSPASPA
22. YSPASPA
23. YSPASPA
24. YSSAGAQ PAANAGA
25. YSPASPA
26. YSPASPV

Fig. S1. Vertical alignments of model organism and polar yeast CTDs. This arrangement highlights the almost invariant Ser2-Pro3 and Ser5-Pro6 motifs, but the much less-well conserved residues at positions 1, 4 and 7 with in the heptad repeat.

Supplementary Figure S2

A. CTDs sequences arranged by phylogenetic origin

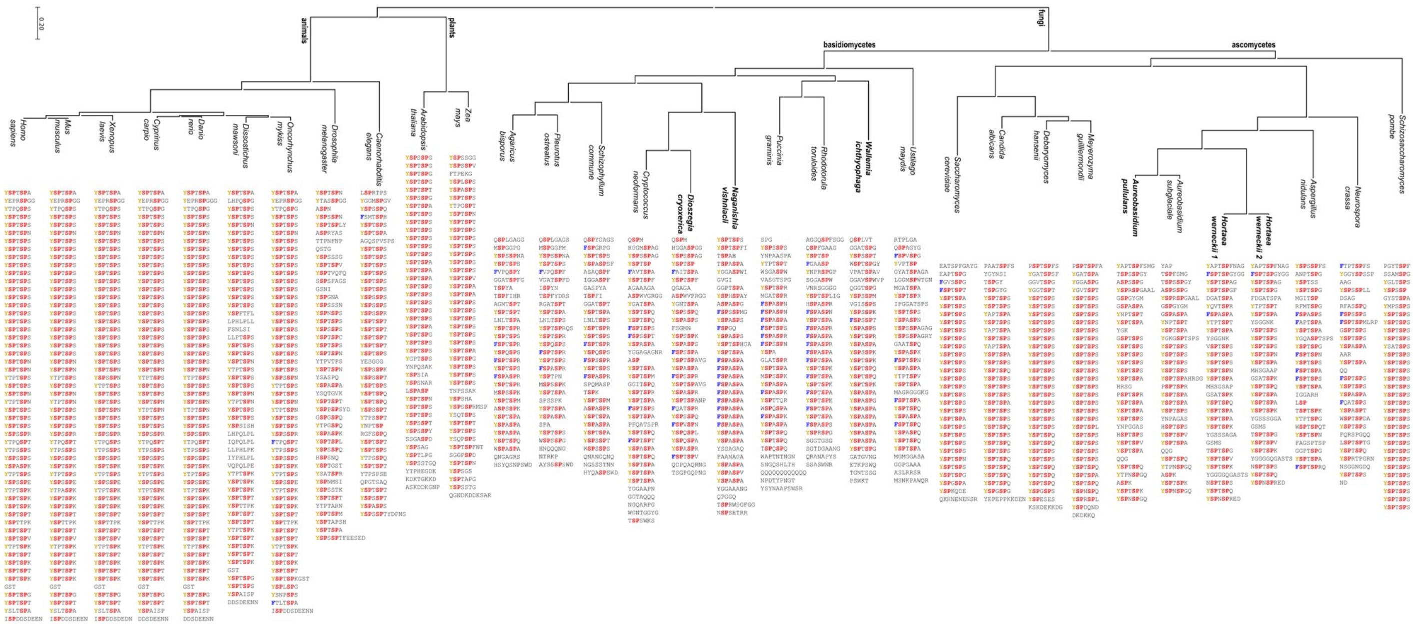

B. Differential conservation of sequences within the CTD heptad repeat

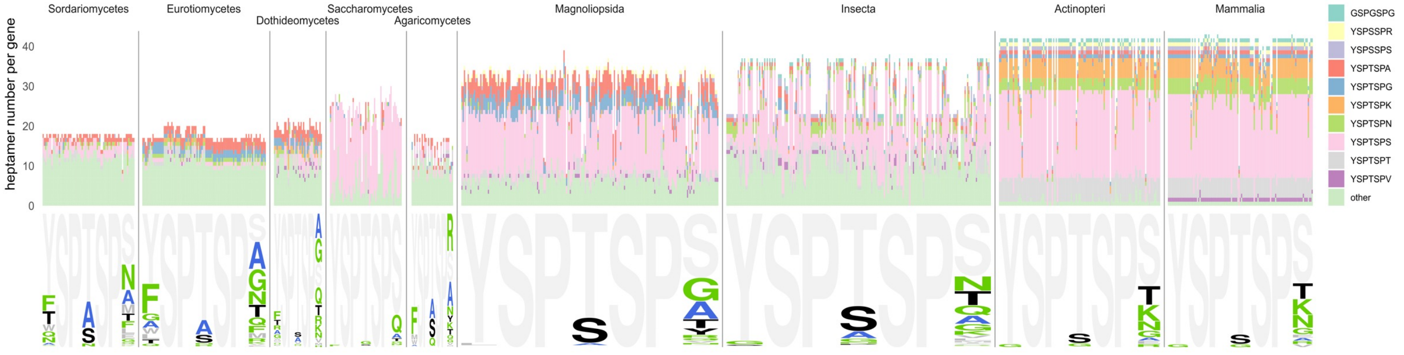

Fig. S2. Expanded display of fungal, plant and metazoan CTD sequences arranged by Rpb1 phylogenic origin. (**A**) The Ser-Pro motifs are highly conserved amongst all these organisms from fungi to humans. The most variability is at position 7, and to a lesser extent position 1 and 4. Interestingly the Y > F substitution at position 1 seems to cluster among the basidiomycetes. In *S. cerevisiae*, uniform Y1F substitution mutants are lethal, but in these sequences, even the most heavily substituted species, *N. vishniacii* and *Rhodotorula. toruloides* still retain some (phosphorylatable) Y1 residues. (**B**) Heptamer consensus sequences among different phyla. Absolute numbers of heptamers in individual organisms, with different heptamers marked with different colors and stacked into columns (one column per genome). Below the chart are relative frequency plot of residues in all identified heptamers of each taxonomic group listed above the column chart; residues of the canonical YSPTSPS are in light grey, major non-canonical residues are colored green, blue and black.

Phylogenetic analysis of Ess1/Pin1 sequences

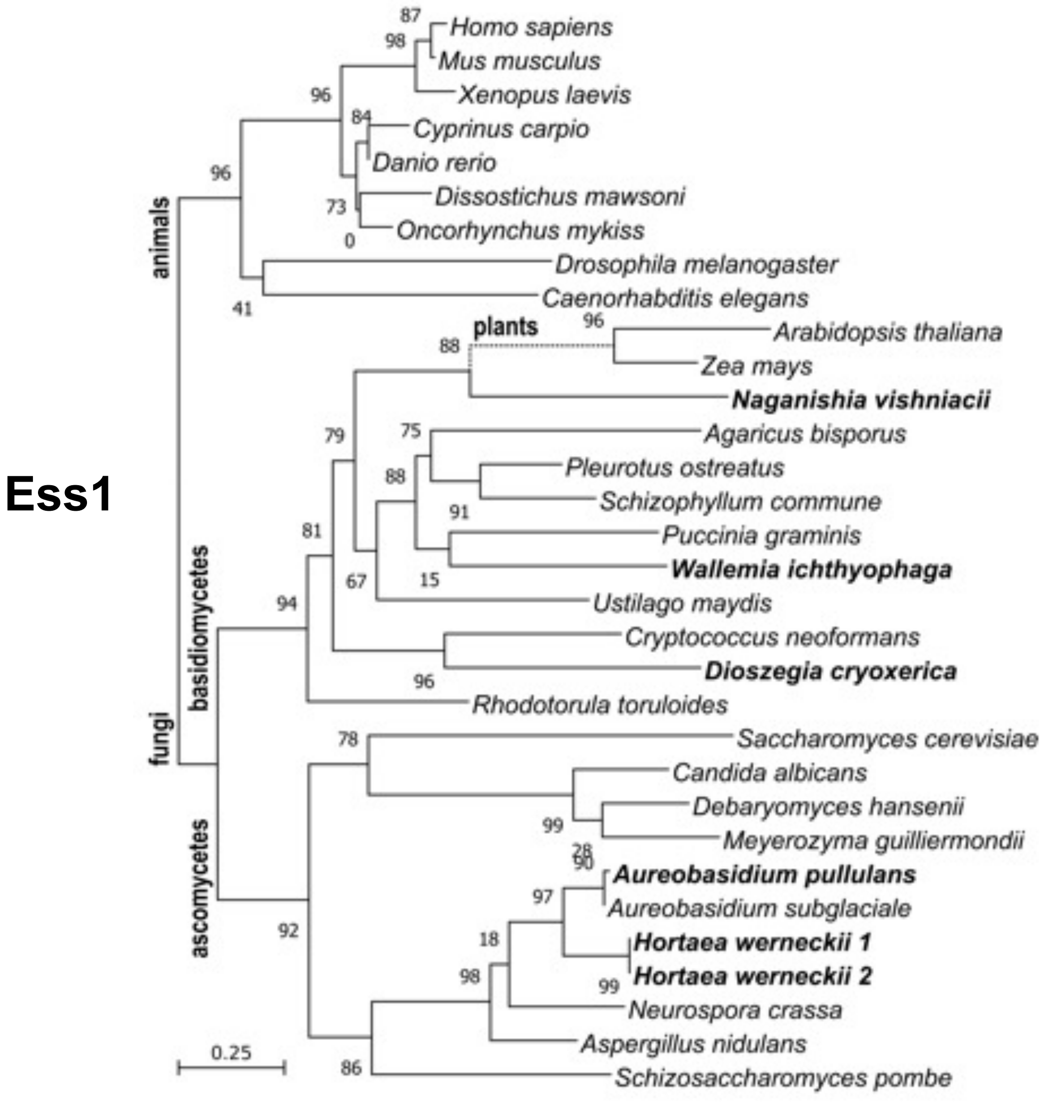

Fig. S3. Maximum likelihood phylogenetic analysis using an alignment of Ess1 coding sequences. Polar yeast species and halophilic fungi studied here are indicated in bold lettering. This shows the relatedness of potential Ess1 orthologs, but due to the small size of Ess1 (170 aa) the information content and thus the confidence of the phylogenic signal is limited. See text for details.

Key features of plasmid constructs

Expression of the *S. cerevisiae RPB1* large subunit of RNAPII with its own CTD replaced by CTDs from Polar yeasts. Expression is driven from the *S. cerevisiae RPB1* promoter (and terminator) in plasmid pRF467 (amp^R CEN, *HIS3*). Two versions were generated, untagged and 3xFLAG epitope-tagged.

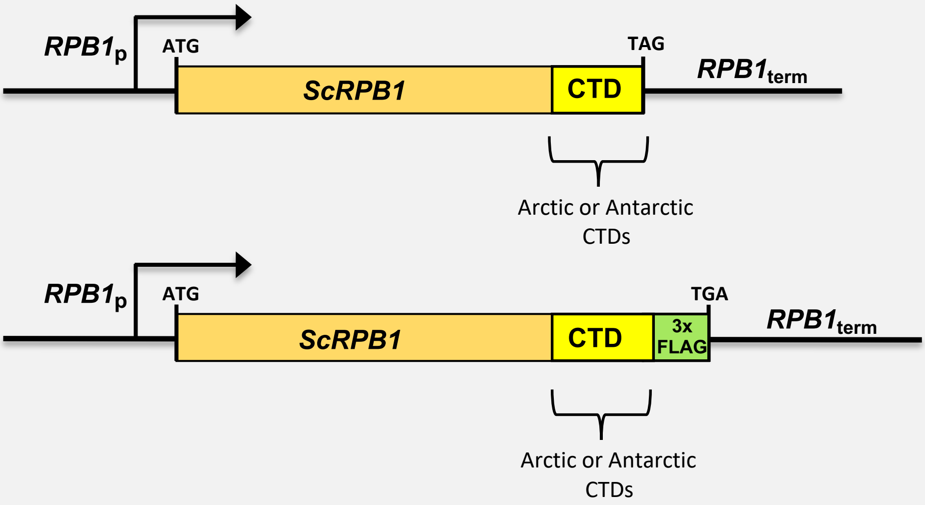

Expression of Polar Ess1s in *S. cerevisiae*. The coding sequences of Polar Ess1s were driven from the constitutive *ADH1* promoter (and terminator) in high-copy plasmid pJGS4-4 (amp^R , 2μM, *TRP1*). An N-terminal epitope tag was included.

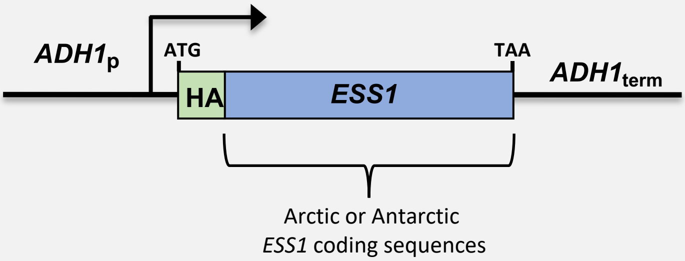

Expression of Polar CTDs as SUMO fusion proteins in *E. coli*. The Polar CTDs were cloned into a pET-SUMO vector and fusion proteins were purified from *E. coli* using the HIS6x-tag by Ni-affinity chromatography that was later removed by thrombin cleavage.

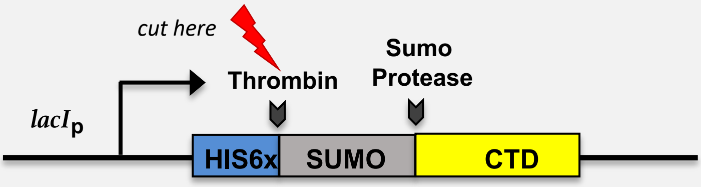

Plasmid-Shuffle Strategy to test complementation by polar CTDs *in vivo.*

Supplementary Figure S4-B

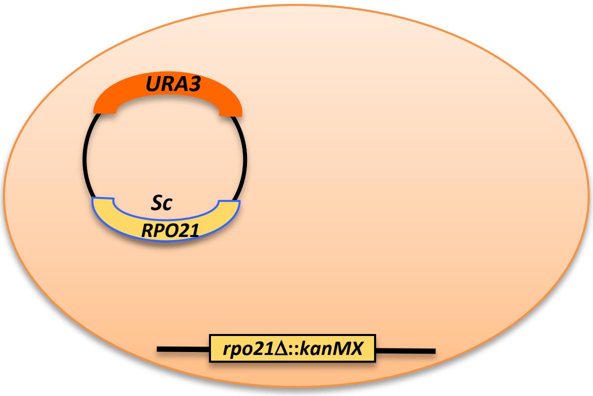

Cells with genomic deletion of the *RPO21* gene (*rpo21*Δ::*kanMX*) covered by plasmid expressing *S. cerevisiae Rpb1*.

Transform with plasmid expressing S. cerevisiae Rpb1(body) with polar CTD "tail"

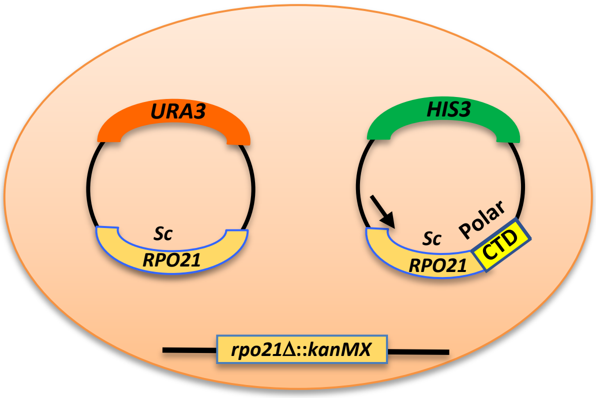

Select for cells that are Ura+ His+

Grow cells in the absence of selection for the ScRPO21 plasmid (CSM-his)

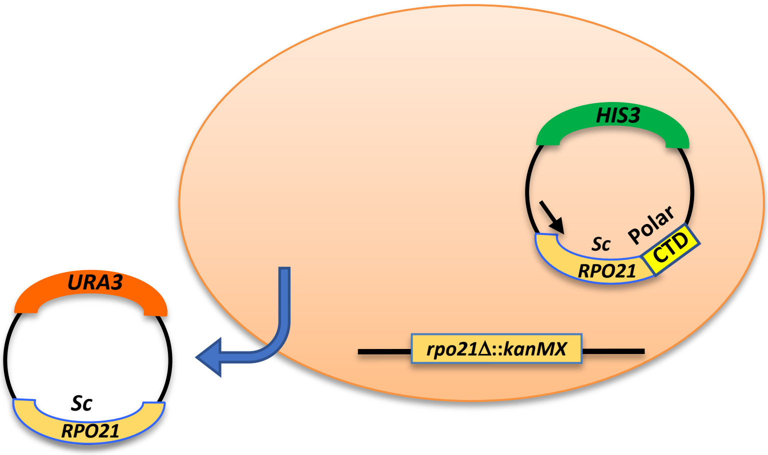

Score for Ura and His phenotype

Cells that are Ura- His+ have lost the covering plasmid (*URA3*) indicating functional complementation of the polar CTD domain. (Cells in which the polar CTD domain gene does not complement will retain the *ScRPO21* plasmid and be Ura+)

Plasmid-Shuffle Strategy to test complementation by polar Ess1s *in vivo*

Supplementary Figure S4-C

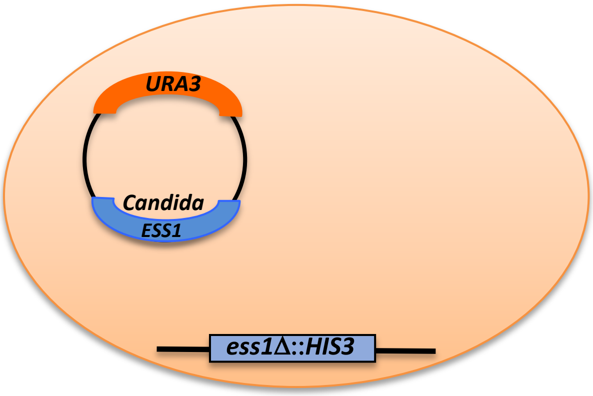

Cells with genomic knockout of the *ESS1* gene (*ess1*Δ::*HIS3*) covered by plasmid expressing *Candida albicans ESS1*.

Transform with plasmid expressing Arctic or Antarctic Ess1

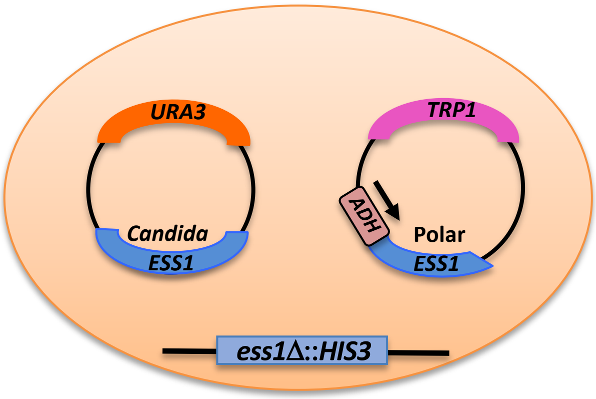

Select for cells that are Ura+ Trp+ (and are His+)

Grow cells in the absence of selection for the Candida ESS1 plasmid (CSM-trp)

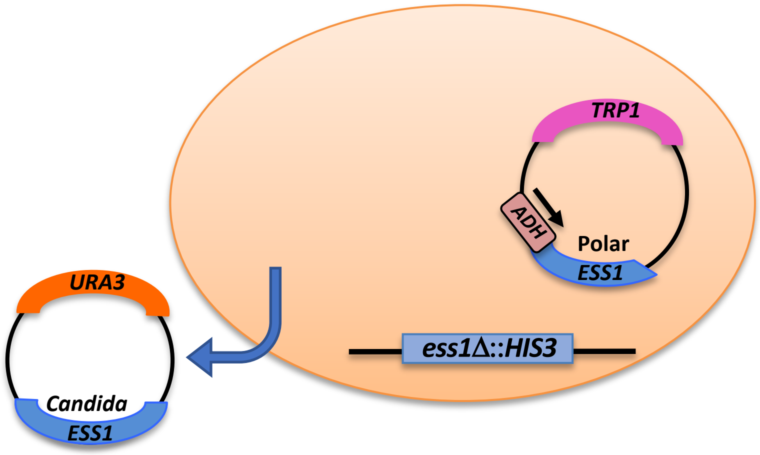

Score for Ura+ and Trp+ phenotype, and confirm genomic knockout *ess1*Δ::*HIS3* (His+)

Cells that are Ura- Trp+ His+ have lost the covering plasmid (*URA3*) indicating functional complementation of the polar *ESS1* gene. (Cells in which the polar *ESS1* gene does not complement will retain the *Candida ESS1* plasmid and be Ura+)

Fig. S4. Diagrams of constructs and yeast genetic strategies used in this study.

(A) Schematic of constructs used to express polar Ess1 enzymes and Rpb1-CTD fusions for complementation assays in *S. cerevisiae* (top, bottom) and SUMO-CTD fusion proteins for purification from *E. coli* (middle). Details given in figure and Materials and Methods. **(B).** Plasmid-shuffle strategy for testing polar CTD function in *S. cerevisiae.* Details given in figure and Materials and Methods. **(C)** Plasmid-shuffle strategy for testing polar Ess1 function in *S. cerevisiae.* Details given in figure and Materials and Methods.

Growth of *S. cerevisiae* host cells that express Rpb1s bearing polar CTDs

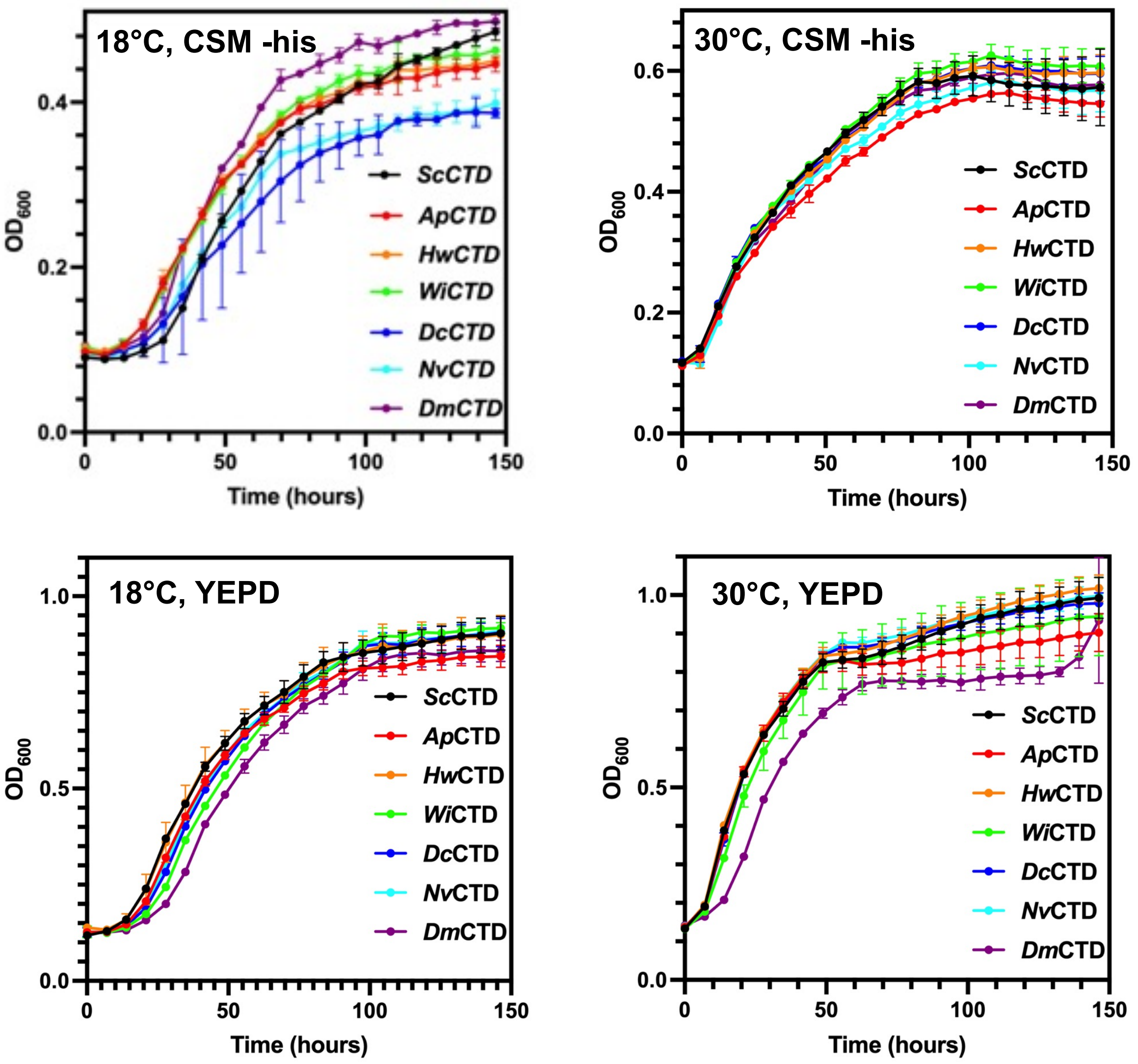

Fig. S5. Growth of *S. cerevisiae* host cells that express CTDs from cold-tolerant yeast species. Cells expressing Rpb1-CTD fusion genes were grown overnight in YEPD liquid (30°C) to mid-log phase (OD_{600nm} 0.5-1.0), diluted to OD_{600nm} = 0.1 (~$2x10^6$ cells/ml) in either rich media (YEPD) or selective media (CMS-his) and growth rates monitored at the indicated temperatures using Tecan instrumentation (see Materials and Methods). Curves are the average of two independent experiments.

Growth of *S. cerevisiae* host cells that express polar Ess1s

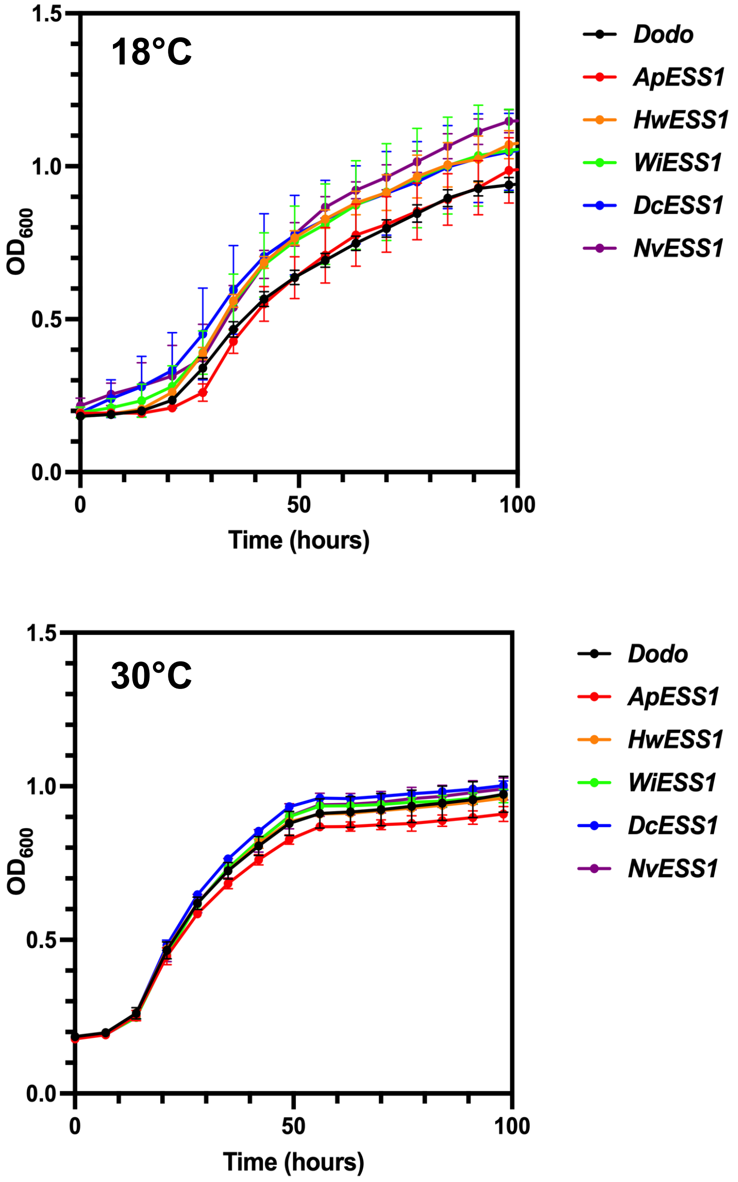

Fig. S6. Growth of *S. cerevisiae* host cells in which the endogenous *ESS1* gene is replaced with the ortholog from cold-tolerant yeast species. Cells were grown overnight in YEPD liquid (30°C) to mid-log phase (OD_{600nm} 0.5-1.0), diluted to OD_{600nm} = 0.1 (~2x10^6 cells/ml) and growth rates monitored at the indicated temperatures using Tecan instrumentation (see Materials and Methods). Expression of the *ESS1* genes was driven from the constitutive ADH1 promoter. *Drosophila* Dodo was used as a positive control. Curves are the average of two independent experiments.

Substitutions (E → P) that may be important for retaining flexibility in the cold-adapted Ess1 enzymes

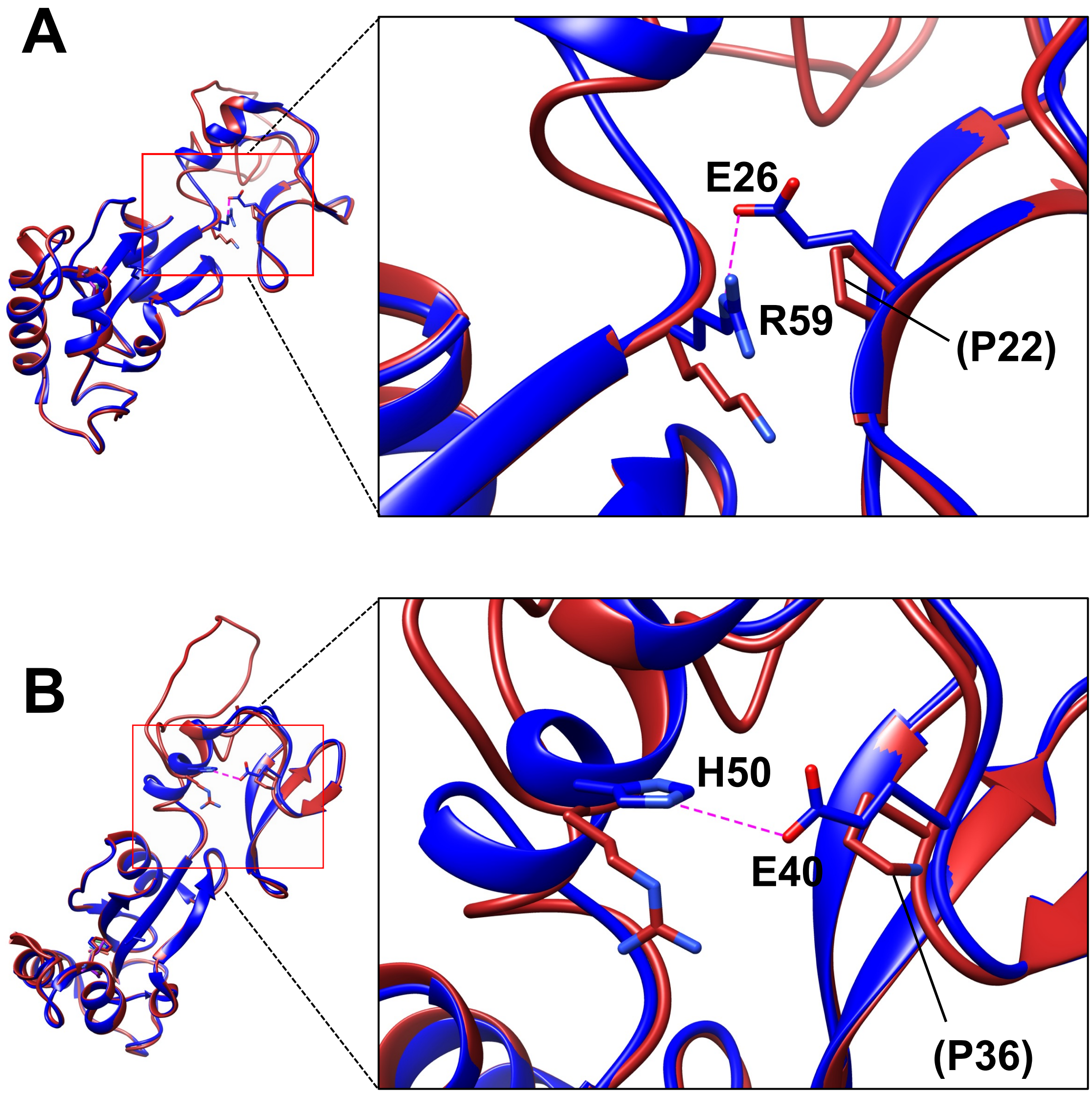

Fig. S7. Substitutions associated with the cold-tolerant Ess1 enzymes. The modeling identifies amino acid substitutions that may be important for folding and enzymatic activity in the cold. ScEss1 (blue) and DcEss1 (red) are shown. (**A**) Structure of ScEss1 showing a slat bridge between E26 and R59, which would be lost with a switch from E26 to Proline in 5/5 of the polar Ess1s (P22 in DcEss1). Loss of this bond would likely weaken the stability of the WW positioning in respect to the prolyl isomerase (catalytic) domain. (**B**) Shows E40 and a potential H-bond to H50. While we don't usually think of histidine as engaging in H-bonds, the planarity of the ring gives the edge a partial positive charge and it is also possible that one of its nitrogens is protonated. Substitution of E40 with proline in 4/5 polar Ess1s (P36 in DcEss1) would remove this interaction, as would alanine in WiEss1.

Supplementary Figure S8

A

C-termini of Polar Rpb1s predicted to be disordered

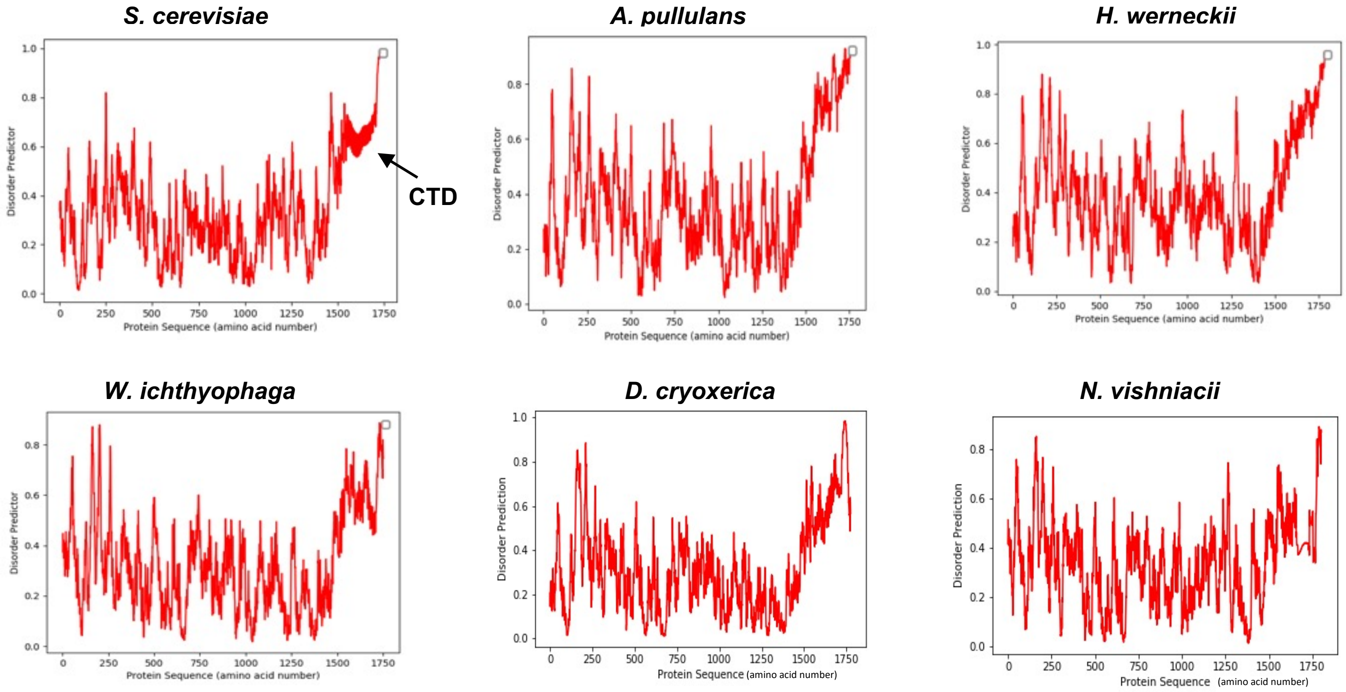

B

Polar CTDs are predicted to undergo phase separation

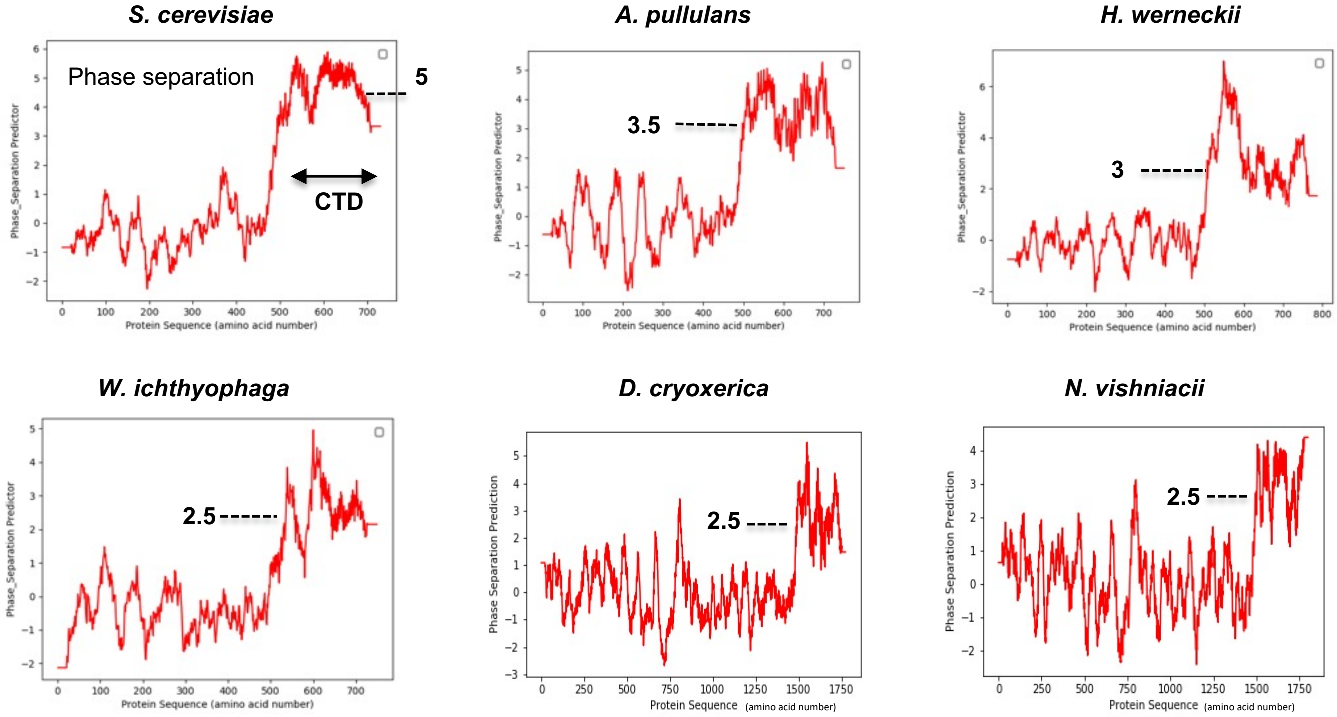

(A) Dosztányi *et al.* (2005) UPred: web server for the prediction of intrinsically unstructured regions of proteins based on estimated energy content. Bioinformatics (2005) **21**, 3433-3434.

(B) Vernon, R. M. *et al*. Pi-Pi contacts are an overlooked protein feature relevant to phase separation. eLife 7, e31486 (2018).

Fig. S8. The Rpb1 subunits of RNAPII from polar yeasts are predicted to have disordered carboxy termini that can undergo LLPS. (A) The predicted Rpb1 coding sequences were analyzed using the UPred program (ref. 46) to identify potential IDRs. In each case the carboxy-terminal ~150 residues show the highest value for predicted disorder. *S. cerevisiae* Rpb1 was used as a control. **(B)** The C-terminal 700 amino acids of the predicted Rpb1 coding sequences were analyzed for propensity to phase separate using a predictor program trained on pi-interactions (ref. 47). Pi-Pi interactions seem to promote LLPS and can occur between a number of peptide moieties, including sidechains of aromatic residues (Y,F,W,H) or via mainchain interactions (from S,T, P) (ref. 47). Note the CTD contains an abundance of such residues (YSPTPSP)

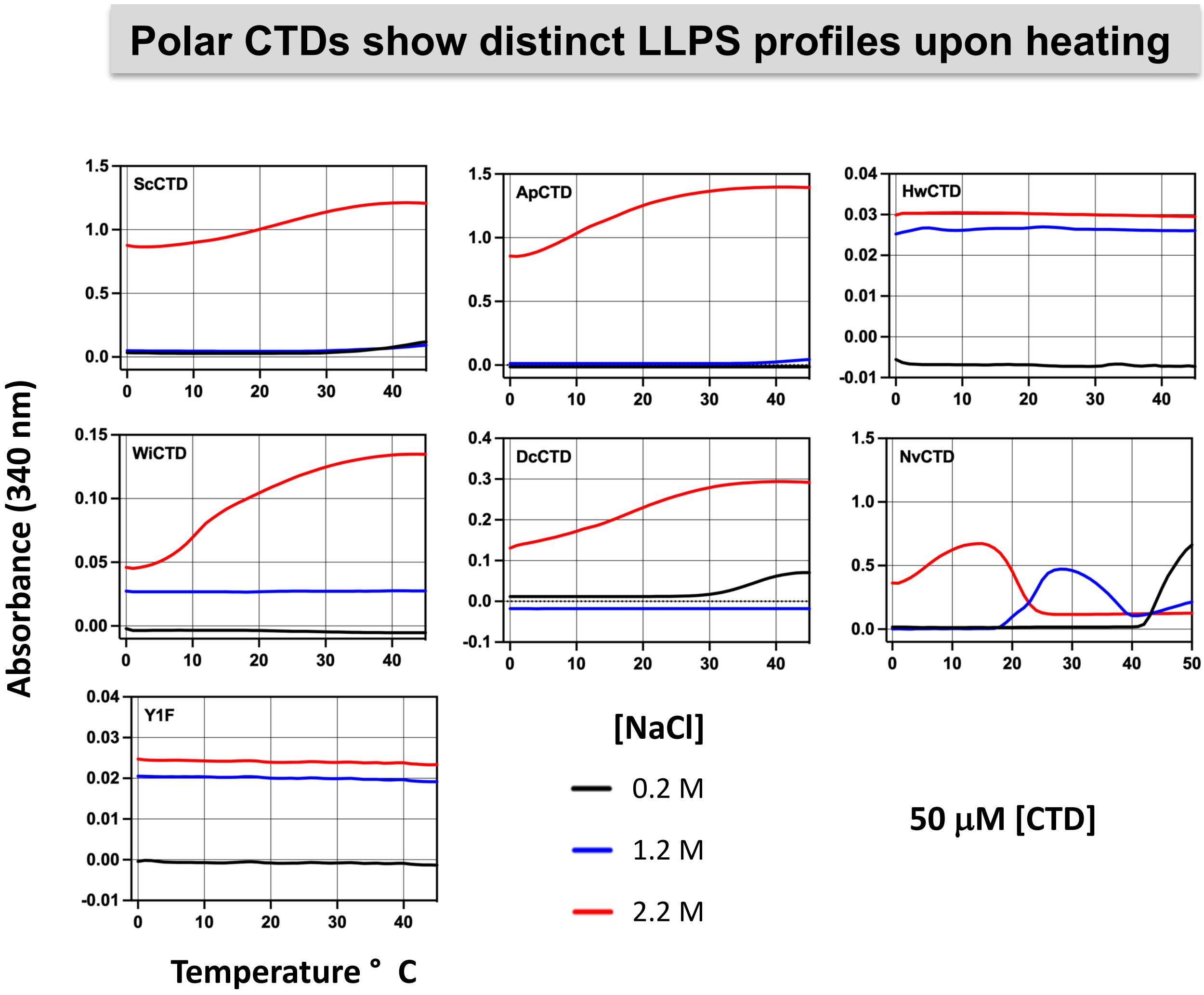

Fig. 9. Light scattering assays reveal distinct LLPS profiles for different CTDs under heating conditions. Each panel represents scattering profiles of the indicated SUMO-CTD fusion protein (50 µM) at three different salt concentrations; ~ 0.2 M, 1.2 M and 2.2 M (220 mM KCl + 0, 1.0 M and 2.0 M NaCl, respectively). Light scattering was continuously monitored at 340 nm as the purified proteins were heated from ~0°C to >40°C. At this concentration, only NvCTD seems to undergo a dual LCST/UCST phase separation (see text). HwCTD and Y1F ScCTD did not show any detectable LLPS under these conditions.

Polar CTDs show distinct LLPS profiles upon cooling

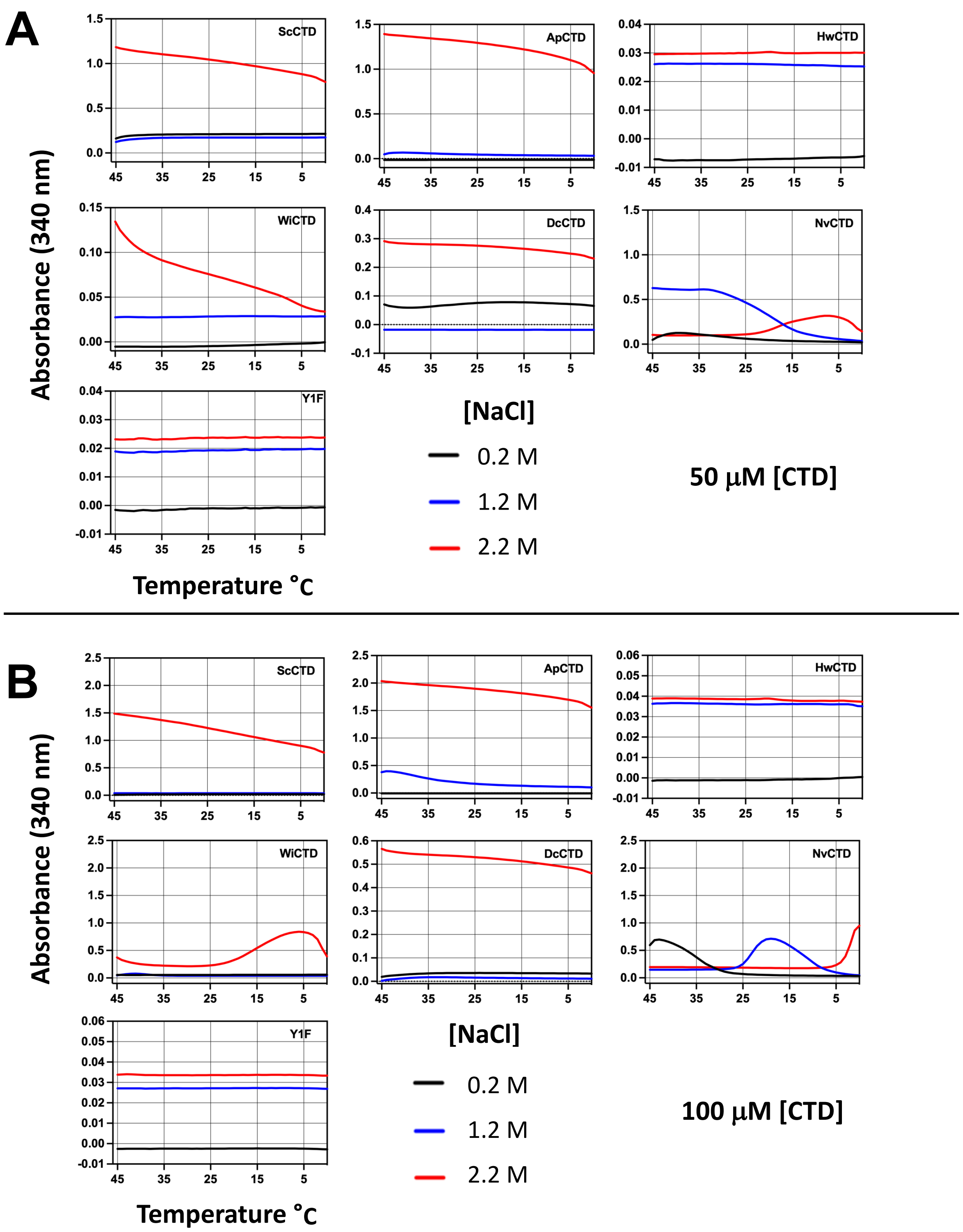

Fig. 10. Light scattering assays reveal distinct LLPS profiles for different CTDs under cooling conditions. Each panel represents light scattering profiles of the indicated SUMO-CTD fusion protein at three different salt concentrations; ~ 0.2 M, 1.2 M and 2.2 M (220 mM KCl + 0, 1.0 M and 2.0 M NaCl, respectively). (**A**) SUMO-CT fusion protein concentrations were 50 µM or (**B**) 100 µM. Light scattering was continuously monitored at 340 nm as the purified proteins were cooled from 45°C to <5°C. HwCTD and Y1F ScCTD did not show any LLPS under these conditions. Note that the cooling profiles are not the mirror image of the heating profiles.

Localization and expression of polar CTDs (18°C)

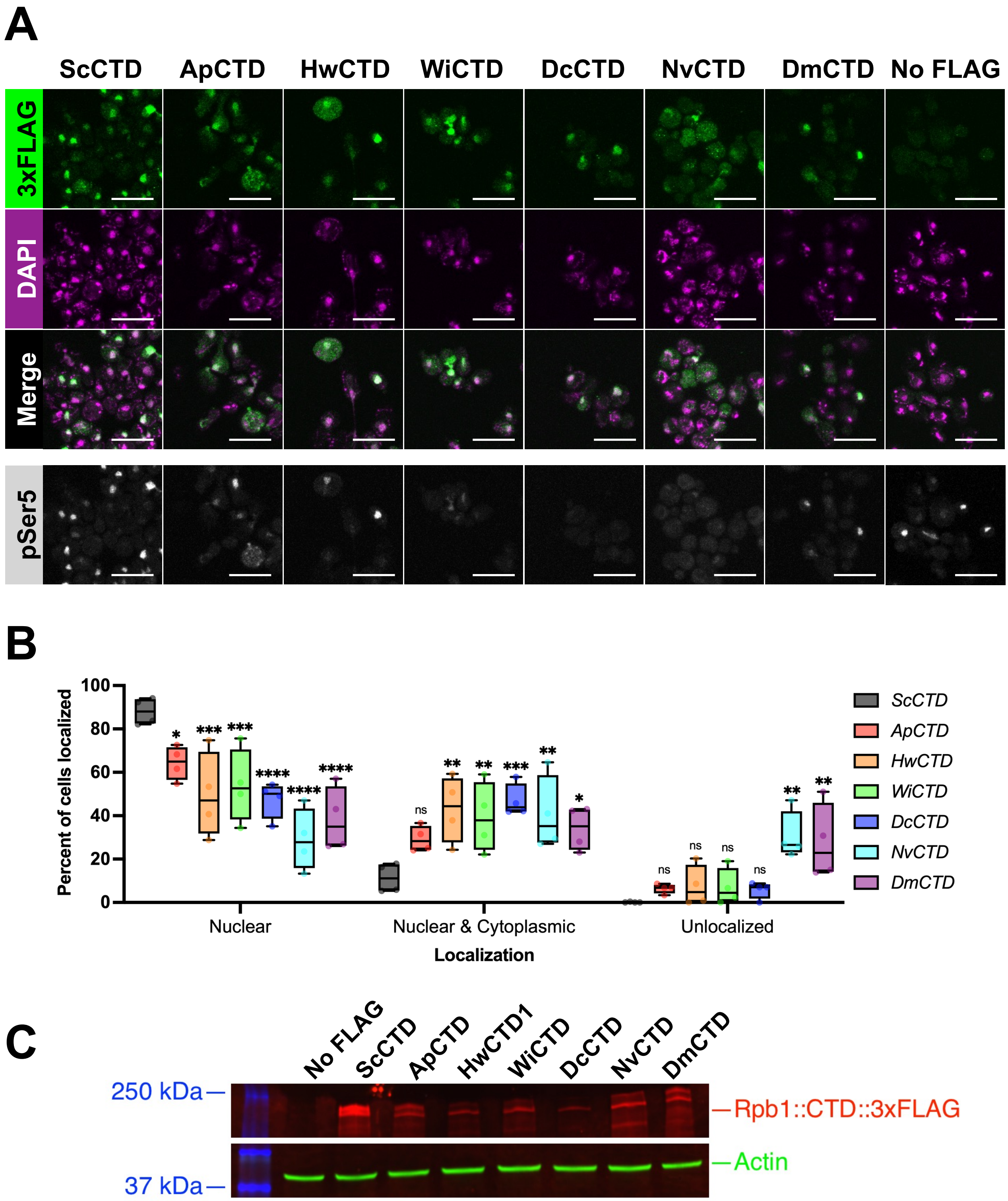

Fig. S11. Polar CTDs fail to localize the Rpb1 subunit to nuclear discrete foci as does the wild-type ScCTD. Immunofluorescent staining of fixed *S. cerevisiae* cells expressing the indicated polar yeast CTD. **(A)** Cells grown at 18°C in CSM-his liquid medium were fixed, and double immunostained with rabbit anti-FLAG to detect the C-terminally FLAG-tagged Rpb1-CTD_n fusions and rat monoclonal anti-pSer2-CTD antibodies (3E10) to determine the levels of Ser2 phosphorylation within the CTD. Nuclei are identified by DAPI staining. Scale bars are 10 μM. **(B)** Subcellular localization of (Rpb1::CTD::3xFLAG) was scored as being localized to the (i) nucleus, (ii) nucleus and cytoplasm, or (iii) unlocalized (dispersed with no distinct nuclear accumulation). Experiments were performed in biological quadruplicate. None of the cold-tolerant CTDs directed Rpb1 exclusively to the nucleus in *S. cerevisiae* cells as does the "wild-type" Sc-CTD under these conditions. Data are represented as interleaved box-and-whisker plots. Statistical significance was calculated by ordinary two-way ANOVA with Dunnett's multiple comparisons test with a single pooled variance using GraphPad Prism 9.4, with *P* values of <.05 (*), <.01 (**), <.001 (***), <.0001 (****), and not significant (ns) indicated. n = 703 (*Sc*CTD), 372 (*Ap*CTD), 282 (*Hw*CTD), 313 (*Wi*CTD), 227 (*Dc*CTD), 313 (*Nv*CTD), and 258 (*Dm*CTD). (**C**) Western analysis to monitor expression levels of the polar yeast Rpb1-CTD constructs. Cells were grown at 18°C and fractionated extracts reacted with anti-FLAG plus anti-actin antibodies. The No FLAG lane contains protein extract from cells expressing untagged-ScCTD as a negative control. DmCTD is a positive control expressing a *D. melanogaster* CTD fusion. Note that expression levels seem to be reduced somewhat in all the CTDs compared to the ScCTD control. And for some (Hw, Wi, Dc) the upper band, likely to represent the phospho-forms of the CTD, is greatly diminished.

Supplementary Figure S12

Localization of polar CTDs

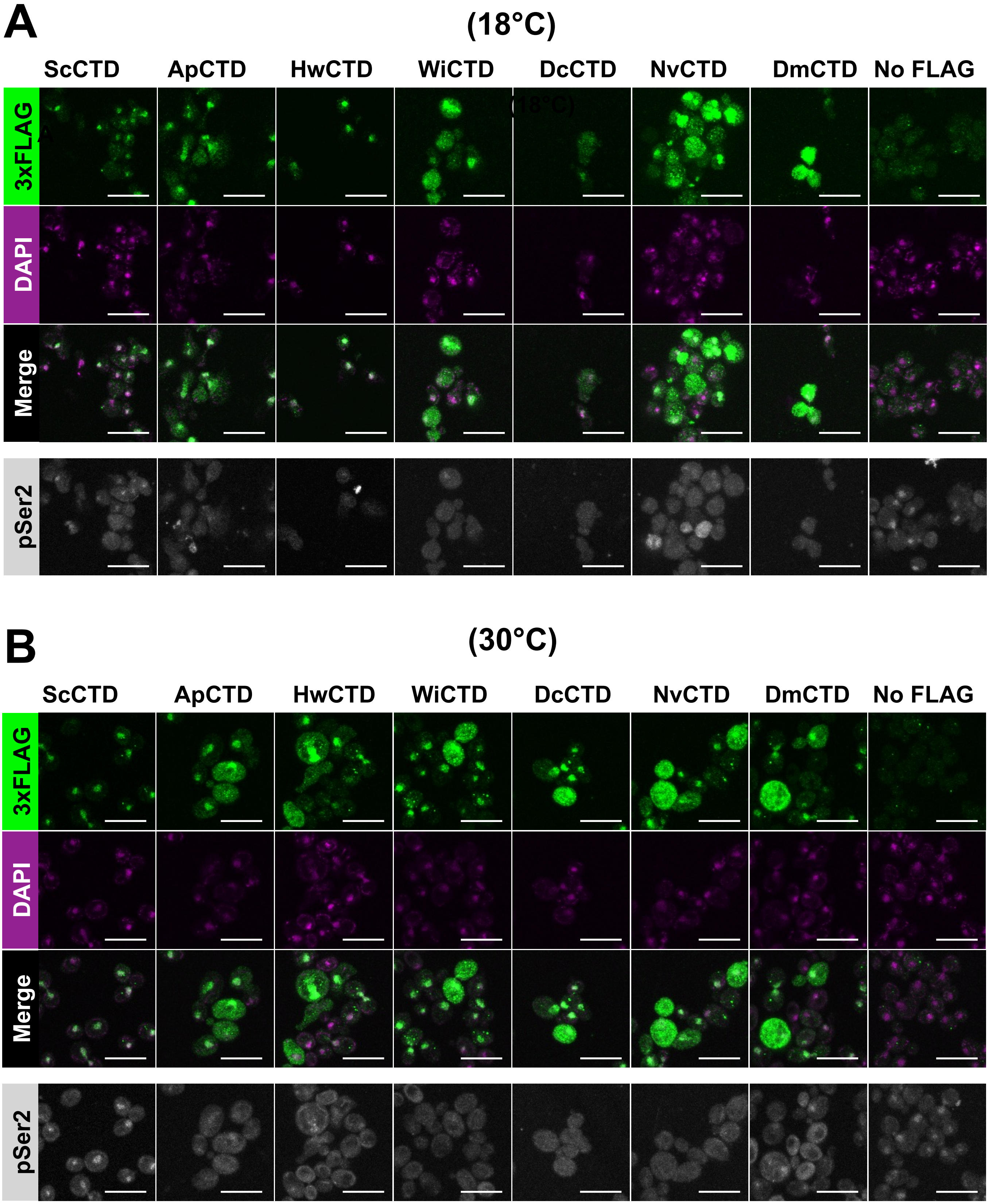

Fig. S12. Polar CTDs fail to localize the Rpb1 subunit to nuclear discrete foci as does the wild-type ScCTD. Immunofluorescent staining of fixed *S. cerevisiae* cells expressing the indicated polar yeast CTD. **(A)** Cells grown at 18°C in CSM-his liquid medium were fixed, and double immunostained with rabbit anti-FLAG to detect the C-terminally FLAG-tagged Rpb1-CTD_n fusions and rat monoclonal anti-pSer2-CTD antibodies (3E10) to determine the levels of Ser2 phosphorylation within the CTD. pSer2 levels appear to be reduced relative to control cells subject to certain caveats (see main text). Nuclei are identified by DAPI staining. **(B)** Cells were grown at 30°C fixed and DAPI-stained as in (A) above. Scale bars are 10 μM.

Table S1. Cold-adapted and salt-tolerant fungal strains used in this study

Species name	Location	Phylum (Class)	Notable features	refs
Aureobasidium pullulans	Kongsvegen Glacier, Spitsbergen Svalbard, Norway	Ascomycota (Dothideomycetes)	Ubiquitous and polyextremotolerant. Used in biotechnology and as a biocontrol agent in agriculture; strain EXF-3645 isolated from glacial ice	Gostincar *et al.* BMC Genomics *15*, 549 (2014) [ref 27]
Hortaea werneckii	Sečovlje saltern, Slovenia	Ascomycota (Dothideomycetes)	Grows over almost the entire salinity (NaCl) range; strain EXF-2000 isolated from hypersaline water	Perini *et al*., Sci Rep 9, 20230 (2019) [ref 29]
Wallemia ichthyophaga	Sečovlje saltern, Slovenia	Basidiomycota (Wallemiomycetes)	Most halophilic fungus known, requires low water activity, grows in saturated NaCl solution; strain EXF-994 isolated from hypersaline water	Zalar *et al*., *Antonie van Leeuwenhoek 87*, 311 (2005) [ref 31]
Dioszegia cryoxerica	Taylor Valley, Victoria Land, Antarctica	Basidiomycota (Tremellomycetes)	May be unique to Southern Victoria Land Antarctica and grows best between 10°C and 15°C but not above 20°C.	Connell *et al*., Int J. Syst. Evol Microbiol, *60*, 1466 (2010) [ref 33]
Naganishia vishniacii	Wright Valley, Victoria Land, Antarctica	Basidiomycota (Tremellomycetes)	May be unique to McMurdo Dry Valleys grows at 4°C and −3°C, but not at or above 21°C.	Nizovoy *et al.*, FEMS Yeast Res. *21*, 1 (2021) [ref 34]

Table S2. Initial CTD LLPS Conditions

SUMO-CTD fusions tested: 7 species, 1 mutant
Buffer conditions: 220 mM KCl, 20 mM Hepes pH7.4
5 [NaCl] added: (0, 0.5, 1.0, 1.5, 2.0)
3 temps: (4°C, RT, 37°C)

Ratings: - = clear (no phase separation)
+/- = slightly cloudy (mild-moderate phase separation)
\+ = noticeably cloudy (phase separation evident)

**Species**		**0.0M NaCl**	**0.5M NaCl**	**1.0M NaCl**	**1.5M NaCl**	**2.0M NaCl**
S.c.	**4°C**	-	-	-	-	-/+
	**RT**	-	-	-	-	+
	**37°C**	-	-	-	-	-
A.p.	**4°C**	-	-	-	-	-
	**RT**	-	-	-	-	-
	**37°C**	-	-	-	-	-/+
H.w.	**4°C**	-	-	-	-	-/+
	**RT**	-	-	-	-/+	+
	**37°C**	-	-	-	+	+
W.i.	**4°C**	-	-	-	+	+
	**RT**	-	-	-	+	+
	**37°C**	-	-	-	-/+ *	-/+
D.c.	**4°C**	-	-	-/+	+	+
	**RT**	-	-	-	-	-/+
	**37°C**	-	-	-	-	+
N.v.	**4°C**	-	-	-/+	+	+
	**RT**	-	-	-	-	-
	**37°C**	+	-	-	-	-
D.m.	**4°C**	-/+	+	-/+	-/+	-/+
	**RT**	-/+	-/+	-/+	-/+	-/+
	**37°C**	-	-/+	-/+	-/+	-/+
**Y1F**	**4°C**	-	-	-	-	-
	**RT**	-	-	-	-	-
	**37°C**	-	-	-	-	-

**Cloudiness here may be an artefact of time it takes to take photo*

Table S3. PCR conditions used in this study.

Fragment(s)	Conditions			Cycles	Primers	Template	Purpose
	Step	Temp	Time				
Ap ESS1<br>Wi ESS1	initial denaturation	98°C	30 "	25	Ap: UP0182 (F), UP0192 (R)<br><br>Wi: UP0173 (F), UP0190 (R)	PCR fragment	Add pJGS4-4 vector overhangs to ESS1 fragment
	denaturation	98°C	10 "				
	annealing	63°C	30 "				
	extension	72°C	16 "				
	final extension	72°C	5 '				
	hold	4°C	∞				
Dm ESS1	initial denaturation	98°C	30 "	25	UP0186 (F), UP0193 (R)	PCR fragment	Add pJGS4-4 vector overhangs to ESS1 fragment
	denaturation	98°C	10 "				
	annealing	67°C	30 "				
	extension	72°C	15 "				
	final extension	72°C	5 '				
	hold	4°C	∞				
Hw ESS1	initial denaturation	98°C	30 "	25	UP0178 (F), UP0191 (R)	PCR fragment	Add pJGS4-4 vector overhangs to ESS1 fragment
	denaturation	98°C	10 "				
	annealing	64°C	30 "				
	extension	72°C	16 "				
	final extension	72°C	5 '				
	hold	4°C	∞				
Dc ESS1	initial denaturation	98°C	30 "	30	UP0329 (F), UP0330 (R)	gDNA	Amplify Dc ESS1 fragment from genomic DNA
	denaturation	98°C	10 "				
	annealing	70°C	30 "				
	extension	72°C	31 "				
	final extension	72°C	5 '				
	hold	4°C	∞				
Nv ESS1	initial denaturation	98°C	30 "	30	UP0331 (F), UP0332 (R)	gDNA	Amplify Nv ESS1 fragment from genomic DNA
	denaturation	98°C	10 "				
	annealing	67°C	30 "				
	extension	72°C	30 "				
	final extension	72°C	5 '				
	hold	4°C	∞				
Dc ESS1 exons 1, 2, 3	initial denaturation	98°C	30 "	20	exon 1: UP0353 (F), UP0342 (R)<br>exon 2: UP0343 (F), UP0344 (R)<br>exon 3: UP0345 (F), UP0354 (R)	PCR fragment	Amplify Dc ESS1 exons with pJGS4-4 overhangs
	denaturation	98°C	10 "				
	annealing	72°C	30 "				
	extension	72°C	31 "				
	final extension	72°C	5 '				
	hold	4°C	∞				
Nv ESS1 exons 1, 2, 3	initial denaturation	98°C	30 "	20	exon 1: UP0355 (F), UP0348 (R)<br>exon 2: UP0349 (F), UP0350 (R)<br>exon 3: UP0351 (F), UP0356 (R)	PCR fragment	Amplify Nv ESS1 exons with pJGS4-4 overhangs
	denaturation	98°C	10 "				
	annealing	72°C	30 "				
	extension	72°C	31 "				
	final extension	72°C	5 '				
	hold	4°C	∞				
Dc ESS1 exons 1, 2	initial denaturation	98°C	30 "	20	UP0353 (F), UP0344 (R)	n/a	PCR SOEing to join Dc ESS1 exons 1 and 2 together
	denaturation	98°C	10 "				
	annealing	72°C	10 "				
	extension	72°C	10 "				
	final extension	72°C	5 '				
	hold	4°C	∞				

Nv ESS1 exons 1, 2	initial denaturation	98°C	30 "	20	UP0355 (F), UP0350 (R)	n/a	PCR SOEing to join Nv ESS1 exons 1 and 2 together
	denaturation	98°C	10 "				
	annealing	72°C	10 "				
	extension	72°C	10 "				
	final extension	72°C	5 '				
	hold	4°C	∞				
Dc ESS1 exons 1, 2, 3	initial denaturation	98°C	30 "	20	UP0353 (F), UP0366 (R)	n/a	PCR SOEing to add exon 3 to Dc ESS1 exons 1 and 2
	denaturation	98°C	10 "				
	annealing	72°C	10 "				
	extension	72°C	20 "				
	final extension	72°C	5 '				
	hold	4°C	∞				
Nv ESS1 exons 1, 2, 3	initial denaturation	98°C	30 "	20	UP0355 (F), UP0367 (R)	n/a	PCR SOEing to add exon 3 to Nv ESS1 exons 1 and 2
	denaturation	98°C	10 "				
	annealing	72°C	10 "				
	extension	72°C	20 "				
	final extension	72°C	5 '				
	hold	4°C	∞				
Nv ESS1	initial denaturation	98°C	30 "	30	UP0368	plasmid	Remove suspected intron sequence from Nv ESS1
	denaturation	98°C	10 "				
	annealing	64°C	10 "				
	extension	72°C	5 '				
	final extension	72°C	5 '				
	hold	4°C	∞				
Ap, Hw, Wi, Dc, Nv ESS1's	initial denaturation	98°C	30 "	25	Ap: UP0384, UP0385<br>Hw: UP0386, UP0387<br>Wi: UP0388, UP0389<br>Dc: UP0390, UP0391<br>Nv w/ "intron": UP0392, UP0393<br>Nv w/o "intron": UP0392, UP0393	plasmid	Amplify ESS1 from pJGS4-4 for cloning into pet28b+ (to produce ESS1 protein)
	denaturation	98°C	10 "				
	annealing	63°C	15 "				
	extension	72°C	19 "				
	final extension	72°C	5 '				
	hold	4°C	∞				
Sc, Hw_2, Wi CTDs	initial denaturation	98°C	30 "	30	Sc: UP0265, UP0266<br>Hw_2: UP0271, UP0272<br>Wi: UP0273, UP0274	genomic DNA for Sc, cDNA for Hw, Wi	Amplify Arctic* (+ Sc control) CTDs out of cDNA for cloning into pET-SUMO vector
	denaturation	98°C	10 "				
	annealing	72°C	15 "				
	extension	72°C	30 "				
	final extension	72°C	5 '				
	hold	4°C	∞				
Ap, Hw_1 CTDs	initial denaturation	98°C	30 "	30	Hw_1: UP0269, UP0270<br>Ap: UP0267, UP0268	cDNA	Amplify Arctic CTDs out of cDNA for cloning into pET-SUMO vector
	denaturation	98°C	10 "				
	annealing	69°C (cycles 1-2)<br>72°C (cycles 3-30)	15 "				
	extension	72°C	30 "				
	final extension	72°C	5 '				
	hold	4°C	∞				
Sc, Ap, Hw_1, Hw_2, Wi CTDs	initial denaturation	98°C	30 "	25	Sc: UP0307, UP0308<br>Ap: UP0309, UP0310<br>Hw_1: UP0311, UP0312<br>Hw_2: UP0313, UP0314	plasmid	Amplify Arctic CTDs out of pET-SUMO vectors for cloning into pFR467
	denaturation	98°C	10 "				
	annealing	67°C (cycles 1-2)<br>72°C (cycles 3-25)	15 "				

	extension	72°C	20 "		Wi: UP0315, UP0316		
	final extension	72°C	5 '				
	hold	4°C	∞				
Dc_1, Dc_2 CTDs	initial denaturation	98°C	30 "	35	Dc_1: UP0319, UP0320<br>Dc_2: UP0321, UP0322	gDNA	Amplify Antarctic** CTDs out of genomic DNA for cloning into pET-SUMO vector
	denaturation	98°C	10 "				
	annealing	61°C (cycles 1-2)<br>72°C (cycles 3-25)	15 "				
	extension	72°C	20 "				
	final extension	72°C	5 '				
	hold	4°C	∞				
Nv CTD exons 2 & 3	initial denaturation	98°C	30 "	35	exon 2: UP0361, UP0362<br>exon 3: UP0363, UP0326	gDNA	Amplify Nv CTD exons 2 & 3 out of genomic DNA for cloning into pET-SUMO vector. Exon 1 was made by a 73 bp ssDNA, to be added to vector via HiFi assembly
	denaturation	98°C	10 "				
	annealing	62°C (cycles 1-2)<br>72°C (cycles 3-25)	15 "				
	extension	72°C	20 "				
	final extension	72°C	5 '				
	hold	4°C	∞				
Nv CTD exons 2 & 3	initial denaturation	98°C	30 "	20	UP0361, UP0326	PCR fragments	PCR SOEing of Nv CTD exons 2 & 3.
	denaturation	98°C	10 "				
	annealing	72°C	20 "				
	extension	72°C	30 "				
	final extension	72°C	5 '				
	hold	4°C	∞				
Dc_1, Dc_2, Nv CTDs	initial denaturation	98°C	30 "	30	Dc_1: UP0357, UP0358<br>Dc_2: UP0359, UP0360<br>Nv: UP0364, UP0365	plasmid	Amplify Antarctic CTDs out of pET-SUMO for cloning into pFR467
	denaturation	98°C	10 "				
	annealing	72°C	15 "				
	extension	72°C	25 "				
	final extension	72°C	2 '				
	hold	4°C	∞				
Dc_1 CTD	initial denaturation	98°C	30 "	35	UP0406, UP0407	gDNA	Prior attempts to amplify Dc_1 CTD using overhangs resulted in Dc_2 being amplified. So nested PCR was used to amplify Dc_1 for cloning into pFR467.
	denaturation	98°C	10 "				
	annealing	72°C	15 "				
	extension	72°C	20 "				
	final extension	72°C	2 '				
	hold	4°C	∞				
Dc_1 CTD	initial denaturation	98°C	30 "	25	UP0357, UP0358	PCR fragment	Add pFR467 overhangs to Dc_1 CTD for HiFi assembly
	denaturation	98°C	10 "				
	annealing	66°C (cycles 1-2)<br>72°C (cycles 3-25)	15 "				
	extension	72°C	20 "				
	final extension	72°C	5 '				
	hold	4°C	∞				
Dm CTD	initial denaturation	98°C	30 "	35	UP0380, UP0381	gDNA	Amplify Dm CTD from genomic DNA for cloning
	denaturation	98°C	10 "				
	annealing	72°C	15 "				

	extension	72°C	29 "				into pET-SUMO vector
	final extension	72°C	5 '				
	hold	4°C	∞				
Y1F CTD	initial denaturation	98°C	30 "	25	UP0382, UP0383	plasmid	Amplify Y1F CTD from pFR511 plasmid for cloning into pET-SUMO vector
	denaturation	98°C	10 "				
	annealing	72°C	15 "				
	extension	72°C	20 "				
	final extension	72°C	2 '				
	hold	4°C	∞				
Dm CTD	initial denaturation	98°C	30 "	25	UP0425, UP0426	plasmid	Amplify Dm CTD from pET-SUMO vector for cloning into pFR467
	denaturation	98°C	10 "				
	annealing	69°C (cycles 1-2)<br>72°C (cycles 3-25)	15 "				
	extension	72°C	28 "				
	final extension	72°C	5 '				
	hold	4°C	∞				
Sc, Ap, Hw_1, Hw_2, Wi, Dc_1, Dc_2, Nv CTDs	initial denaturation	98°C	30 "	25	Sc: UP0307, UP0308<br>Ap: UP0309, UP0445<br>Hw_1: UP0311, UP0446<br>Hw_2: UP0313, UP0447<br>Wi: UP0315, UP0448<br>Dc_1: UP0357, UP0449<br>Dc_2: UP0359, UP0450<br>Nv: UP0364, UP0451	plasmid	Amplification of all polar CTDs from original pFR467 vectors into remade pFR467 vectors designed to express FLAG tag
	denaturation	98°C	10 "				
	annealing	72°C	15 "				
	extension	72°C	10 "				
	final extension	72°C	5 '				
	hold	4°C	∞				
	denaturation	98°C	10 "				
	annealing	70°C (cycles 1-2)<br>72°C (cycles 3-35)	15 "				
	extension	72°C	19 "				
	final extension	72°C	2 '				
	hold	4°C	∞				

Table S4. Oligonucleotides used in this study

Name	Locus	Sequence (5'->3')	Use
UP0171	Ap ESS1 5'-F	GCTACCGGTCTTCCCAGC	Amplify Ap ESS1 from cDNA
UP0172	Ap ESS1 3'-R	CTACTCCAGACGTTCAATCAGG	Amplify Ap ESS1 from cDNA
UP0173	Ap ESS1 5'-F	CCAGATTATGCCGGAGCTACCGGTCTTCC CAGC	Add pJGS4-4 vector overhangs to Ap ESS1 fragment
UP0175	Hw ESS1 5'-F	GATCAGGCGACAGGACTACC	Amplify Hw ESS1 from cDNA
UP0176	Hw ESS1 3'-R	TTACTCGAGCCGCTCGATAATATG	Amplify Hw ESS1 from cDNA
UP0178	Hw ESS1 5'-F	CCAGATTATGCCGGAGATCAGGCGACAGG ACTACC	Add pJGS4-4 vector overhangs to Hw ESS1 fragment
UP0180	Wi ESS1 5'-F	ATGACGTGGACGATTAAATTC	Amplify Wi ESS1 from cDNA
UP0181	Wi ESS1 3'-R	TCACTCCAATCTTAATATCAAG	Amplify Wi ESS1 from cDNA
UP0182	Wi ESS1 5'-F	CCAGATTATGCCGGAATGACGTGGACGATT AAATTC	Add pJGS4-4 vector overhangs to Wi ESS1 fragment
UP0184	Dm ESS1 5'-F	CCAGATGCCGAGCAACTACC	Amplify Dm ESS1 from cDNA
UP0185	Dm ESS1 3'-R	CTATGCCTTGCGCAGGATG	Amplify Dm ESS1 from cDNA
UP0186	Dm ESS1 5'-F	CCAGATTATGCCGGACCAGATGCCGAGCAA CTACC	Add pJGS4-4 vector overhangs to Dm ESS1 fragment
UP0190	Ap ESS1 3'-R	CTCGACTAGTTAGTCAGCTCGAGCTACTCC AGACGTTCAATCAGG	Add pJGS4-4 vector overhangs to Ap ESS1 fragment
UP0191	Hw ESS1 3'-R	CTCGACTAGTTAGTCAGCTCGAGTTACTCG AGCCGCTCGATAATATG	Add pJGS4-4 vector overhangs to Hw ESS1 fragment
UP0192	Wi ESS1 3'-R	CTCGACTAGTTAGTCAGCTCGAGTCACTCC AATCTTAATATCAAG	Add pJGS4-4 vector overhangs to Wi ESS1 fragment
UP0193	Dm ESS1 3'-R	CTCGACTAGTTAGTCAGCTCGAGCTATGCC TTGCGCAGGATG	Add pJGS4-4 vector overhangs to Dm ESS1 fragment
ssDNA	n/a	CAACTCCAAGCTCGGAATTCCACCAATGGG TGCTTACCCTTATGATGTGCCAGATTATGC CGGA	Contains EcoRI site + HA tag, bridges the 2 HiFi fragments
UP0265	Sc CTD 5'-F	CAGAGAACAGATTGGTGGTGGCTTTTCTCC AACTTCCCCAACATACTC	Amplify Sc CTD from genomic DNA. Has pET-SUMO vector overhang for HiFi assembly.
UP0266	Sc CTD 3'-R	GTACCCATGGATCCAGTTTATCTGGAATTT TCATTTTCATTATGCTTTTGTTCGTCTTGC	Amplify Sc CTD from genomic DNA. Has pET-SUMO vector overhang for HiFi assembly.
UP0267	Ap CTD 5'-F	CAGAGAACAGATTGGTGGTggtTACAGTGGT GGTCAGAGTCCTGG	Amplify Ap CTD from cDNA. Has pET-SUMO vector overhang for HiFi assembly.
UP0268	Ap CTD 3'-R	GTACCCATGGATCCAGTTTATTGGCCAGGC GAGTTTGGTG	Amplify Ap CTD from cDNA. Has pET-SUMO vector overhang for HiFi assembly.
UP0269	Hw_1 CTD 5'-F	CAGAGAACAGATTGGTGGTGGTTACACAG GCGGCATGTCTCC	Amplify Hw_1 CTD from cDNA. Has pET-SUMO vector overhang for HiFi assembly.
UP0270	Hw_1 CTD 3'-R	GTACCCATGGATCCAGTTTAGTCTTCACGG GGTGAATTAGGC	Amplify Hw_1 CTD from cDNA. Has pET-SUMO vector overhang for HiFi assembly.
UP0271	Hw_2 CTD 5'-F	CAGAGAACAGATTGGTGGTGGTTACACGG GCGGTATGTCGCC	Amplify Hw_2 CTD from cDNA. Has pET-SUMO vector overhang for HiFi assembly.

UP0272	Hw_2 CTD 3'-R	GTACCCATGGATCCAGTTTAGTCTTCACGT GGTGAATTCGGTG	Amplify Hw_2 CTD from cDNA. Has pET-SUMO vector overhang for HiFi assembly.
UP0273	Wi CTD 5'-F	CAGAGAACAGATTGGTGGTGGTTACGGTC AATCGCCATTGGTTACGG	Amplify Wi CTD from cDNA. Has pET-SUMO vector overhang for HiFi assembly.
UP0274	Wi CTD 3'-R	GTACCCATGGATCCAGTTTACGTCTTCCAG CTTGGCCCTG	Amplify Wi CTD from cDNA. Has pET-SUMO vector overhang for HiFi assembly.
UP307	Sc CTD 5'-F	GTCTCCTCACCAGGCCTCGAGGGCTTTTCT CCAACTTCCCCAACATACTC	Amplify Sc CTD from pET-SUMO vector
UP0308	Sc CTD 3'-R	GTAATCTCTAGAGGTCTATCTGGAATTTTCA TTTTCATTATGCTTTTGTTCGTCTTGC	Amplify Sc CTD from pET-SUMO vector
UP0309	Ap CTD 5'-F	GTCTCCTCACCAGGCCTCGAGTACAGTGG TGGTCAGAGTCCTGG	Amplify Ap CTD from pET-SUMO vector
UP0310	Ap CTD 3'-R	GTAATCTCTAGAGGTCTATTGGCCAGGCGA GTTTGGTG	Amplify Ap CTD from pET-SUMO vector
UP0311	Hw_1 CTD 5'-F	GTCTCCTCACCAGGC CTCGAG TACACAGGCGGCATGTCTCC	Amplify Hw CTD sequence #1 from pET-SUMO vector
UP0312	Hw_1 CTD 3'-R	GTAATCTCTAGAGGT CTA GTCTTCACGGGGTGAATTAGGC	Amplify Hw CTD sequence #1 from pET-SUMO vector
UP0313	Hw_2 CTD 5'-F	GTCTCCTCACCAGGC CTCGAG TACACGGGCGGTATGTCGCC	Amplify Hw CTD sequence #2 from pET-SUMO vector
UP0314	Hw_2 CTD 3'-R	GTAATCTCTAGAGGT CTA GTCTTCACGTGGTGAATTCGGTG	Amplify Hw CTD sequence #2 from pET-SUMO vector
UP0315	Wi CTD 5'-F	GTCTCCTCACCAGGC CTCGAG TACGGTCAATCGCCATTGGTTACGG	Amplify Wi CTD from pET-SUMO vector
UP0316	Wi CTD 3'-R	GTAATCTCTAGAGGT CTA CGTCTTCCAGCTTGGCCCTG	Amplify Wi CTD from pET-SUMO vector
UP0317	polar CTD 5'-F	GTGCTGATTATGGTGAAGCC	pFR467 foreword sequencing primer
UP0318	polar CTD 3'-R	GCTCATTTATCGTCATCATC	pFR467 reverse sequencing primer
UP0329	Dc ESS1 5'-F	CCAACGCCTGCCTTTCACTCAAAC	Amplify around Dc ESS1
UP0330	Dc ESS1 3'-R	TCAGGATCCCCTCTGACCCATCTC	Amplify around Dc ESS1
UP0331	Nv ESS1 5'-F	GTTGCCTAGTGACTTGTCGTGTCC	Amplify around Nv ESS1
UP0332	Nv ESS1 3'-R	GGTGTTCGTGAACTCGATTGCAG	Amplify around Nv ESS1
UP0333	Dc_1 CTD 5'-F	GCTACGGGCAGAGTCCCATGCACG	Amplify around Dc CTD sequence #1
UP0334	Dc_1 CTD 3'-R	CGGCGTCGTTCAGCTGGAACAGCT	Amplify around Dc CTD sequence #1
UP0335	Dc_2 CTD 5'-F	GCCTTCTCGCCCATGCAGACCAGC	Amplify around Dc CTD sequence #2
UP0336	Dc_2 CTD 3'-R	GTGTACGTAGGTAGCTGGGGCGTCGC	Amplify around Dc CTD sequence #2
UP0337	Nv CTD 5'-F	CGCAATGACACCGTACGACAATGGC	Amplify around Nv CTD
UP0338	Nv CTD 3'-R	CGTTTTAGGCGTTGGTTGGAAGCG	Amplify around Nv CTD
UP0341	Dc ESS1 Exon 1 5'-F	CAGAGAACAGATTGGTGGT ATGTCAGGCGCAGCACCCAC	Amplify exon 1 of Dc ESS1. Ends are homologous to cloning site of PET-SUMO.
UP0342	Dc ESS1 Exon 1 3'-R	GGACTTCGAGTAATTTGAGCCTCTCGCCAA CTACTAGGCC	Amplify exon 1 of Dc ESS1. 5' end is homologous to ssDNA containing HA tag and cloning site of pJGS4-4. 3' end is homologous to beginning of exon 2.

UP0343	Dc ESS1 Exon 2 5'-F	GGCCTAGTAGTTGGCGAGAGGCTCAAATT ACTCGAAGTCC	Amplify exon 2 of Dc ESS1. 5' overhang is homologous to the end of exon 1. 3' overhang is homologous to the beginning of exon 3.
UP0344	Dc ESS1 Exon 2 3'-R	GGGAGATTGAAGGTTGCTTCCTCGAAGCTA GCCTGCATC	Amplify exon 2 of Dc ESS1. 5' overhang is homologous to the end of exon 1. 3' overhang is homologous to the beginning of exon 3.
UP0345	Dc ESS1 Exon 3 5'-F	GATGCAGGCTAGCTTCGAGGAAGCAACCT TCAATCTCCC	Amplify exon 3 of Dc ESS1. 5' end homologous to end of exon 2. 3' end homologous to XhoI site and 3xStop in pJGS4-4.
UP0346	Dc ESS1 Exon 3 3'-R	GTACCCATGGATCCAGTTTA TATCCGGTTCGCAGTATGAC	Amplify exon 1 of Dc ESS1. Ends are homologous to cloning site of PET-SUMO.
UP0347	Nv ESS1 Exon 2 5'-F	CAGAGAACAGATTGGTGGT ATG TCGAACCCATGGGAAGTCCG	Amplify exon 2 of Nv ESS1. Ends are homologous to cloning site of PET-SUMO.
UP0348	Nv ESS1 Exon 2 3'-R	CGCAGTCGTAATGTTTGGGTTCTTCCAACT CGCTGCC	Amplify exon 2 of Nv ESS1. 5' end homologous to end of exon 3. 3' end homologous to XhoI site and 3xStop in pJGS4-4.
UP0349	Nv ESS1 Exon 3 5'-F	GGCAGCGAGTTGGAAGAACCCAAACATTA CGACTGCG	Amplify exon 3 of Nv ESS1. 5' overhang is homologous to the end of exon 2. 3' overhang is homologous to the beginning of exon 4.
UP0350	Nv ESS1 Exon 3 3'-R	CTCAACGCAAACGTCGCATCCTCGAACGG CTTGTGCATC	Amplify exon 3 of Nv ESS1. 5' overhang is homologous to the end of exon 2. 3' overhang is homologous to the beginning of exon 4.
UP0351	Nv ESS1 Exon 4 5'-F	GATGCACAAGCCGTTCGAGGATGCGACGT TTGCGTTGAG	Amplify exon 4 of Nv ESS1. 5' end homologous to end of exon 3. 3' end homologous to XhoI site and 3xStop in pJGS4-4.
UP0352	Nv ESS1 Exon 4 3'-R	GTACCCATGGATCCAGTTTA TAACCCGTCCTCAGGATCAAATGC	Amplify exon 4 of Nv ESS1. Ends are homologous to cloning site of PET-SUMO.
UP0353	Dc ESS1 Exon 1 5'-F	TTATGATGTGCCAGATTATGCCGGA ATGTCAGGCGCAGCACCCAC	Amplify exon 1 of Dc ESS1. 5' overhang is homologous to ssDNA containing HA tag and cloning site of pJGS4-4 vector. 3' end is homologous to beginning of exon 2.
UP0355	Nv ESS1 Exon 2 5'-F	TTATGATGTGCCAGATTATGCCGGA ATG TCGAACCCATGGGAAGTCCG	Amplify exon 2 of Nv ESS1. 5' end homologous to end of exon 3. 3' end homologous to XhoI site and 3xStop in pJGS4-4.
UP0357	Dc_1 CTD 5'-F	GTCTCCTCACCAGGC CTCGAG TACTCCCCATCCTCCCCG	Amplify DcCTD1 from pET-SUMO to clone into pFR467 by HiFi Assembly
UP0358	Dc_1 CTD 3'-R	GTAATCTCTAGAGGT CTAGCTCTTCCAGCTGGG	Amplify DcCTD1 from pET-SUMO to clone into pFR467 by HiFi Assembly
UP0359	Dc_2 CTD 5'-F	GTCTCCTCACCAGGC CTCGAG TACTCCCCGTCCTCCCCG	Amplify DcCTD2 from pET-SUMO to clone into pFR467 by HiFi Assembly
UP0360	Dc_2 CTD 3'-R	GTAATCTCTAGAGGT CTAACTCTTCCAGCTCGG	Amplify DcCTD2 from pET-SUMO to clone into pFR467 by HiFi Assembly

UP0361	Nv CTD exon 2 5'-F	CCCGTCTTATAGTCCAACGTCACCCTTTAT CACTTCGC	Amplify Exon 2 of NvCTD with 5' homology to Exon1 and 3' homology to exon 3
UP0362	Nv CTD exon 2 3'-R	GTACGCCGGGCTGTGGGA CTGTATGCCGGAGAGGTTG	Amplify Exon 2 of NvCTD with 5' homology to Exon1 and 3' homology to exon 3
UP0363	Nv CTD exon 3 5'-F	CAACCTCTCCGGCATACAGTCCCCACAGC CCGGCGTAC	Amplify exon 3 of NvCTD with 5' homology to exon 2 and 3' homology to pET-SUMO
UP0364	Nv CTD 5'-F	GTCTCCTCACCAGGC CTCGAG TACAGTCCAACTTCCCCG	Amplify NvCTD from pET-SUMO to clone into pFR467 by HiFi Assembly
UP0365	Nv CTD 3'-R	GTAATCTCTAGAGGT CTACCGCCTAGTATGACTTGGC	Amplify NvCTD from pET-SUMO to clone into pFR467 by HiFi Assembly
UP0366	Dc ESS1 Exon 3 3'-R	CTCGACTAGTTAGTCAGCTCGAGCTATCCG GTTCGCAGTATGAC	Amplify exon 3 of Dc ESS1. 5' end homologous to end of exon 2. 3' end homologous to XhoI site and 3xStop in pJGS4-4.
UP0367	Dc ESS1 Exon 4 3'-R	CTCGACTAGTTAGTCAGCTCGAGCTAACCC GTCCTCAGGATCAAATGC	Amplify exon 4 of Nv ESS1. 5' end homologous to end of exon 3. 3' end homologous to XhoI site and 3xStop in pJGS4-4.
UP0368	Nv ESS1 5'-F	GCAGCAACTTGCTGCGCAGGGAGGTGATG AAAAGGTCAGG	Remove possible intron from Nv ESS1 that has been cloned into pJGS4-4. Primer anneals to either side of intron.
UP0369	Nv ESS1 5'-F	CTCGACTAGTTAGTCAGCTCGAGCTAACCC GTCCTCAGGATCAAATGC	Primer spans the junction between possible intron in Nv ESS1 that has been cloned into pJGS4-4, and the sequence just 5' of it. Designed this way to avoid repetitive glutamate codons. Use to confirm loss of intron.
UP0374	Nv ESS1 5'-F	CAAAGAGGGATACGAGAGGC	Reverse transcribe and amplify Nv ESS1
UP0375	Nv ESS1 3'-R	CGCTGCCTTCGTTAATATCC	Reverse transcribe and amplify Nv ESS1
UP0380	Dm CTD 5'-F	CAGAGAACAGATTGGTGGTTATTCGCCAAC GAGTCCGAACTACACGG	Amplify CTD from *D. melanogaster* gDNA with 5' and 3' ends for cloning into PET-SUMO by HiFi Assembly
UP0381	Dm CTD 3'-R	GTACCCATGGATCCAGTTTATCAGTCTTCG CTCTCCTCGAACG	Amplify CTD from Drosophila melanogaster gDNA with 5' and 3' ends for cloning into PET-SUMO by HiFi Assembly
UP0382	Y1F CTD 5'-F	CAGAGAACAGATTGGTGGTGGCTTTAGCC CTACAAGCCCAAC	Amplify Y1F CTD from pFR511 with 5' and 3' ends for cloning into pET-SUMO by HiFi Assembly
UP0383	Y1F CTD 3'-R	GTACCCATGGATCCAGTTTATCTAGAGTTT TCATTTTCATTATGTTTCTGTTCGTCTTG	Amplify Y1F CTD from pFR511 with 5' and 3' ends for cloning into pET-SUMO by HiFi Assembly
UP0384	Ap ESS1 5'-F	GCGGCCTGGTGCCGCGCGGCAGCCATAT GGCTACCGGTCTTCCCAGCG	Amplify Ap ESS1 from pJGS4-4 with 5' and 3' ends for cloning into pET-28b(+) by HiFi Assembly
UP0385	Ap ESS1 3'-R	AGCTTGTCGACGGAGCTCGAATTCGGATC CCTACTCCAGACGTTCAATCAGG	Amplify Ap ESS1 from pJGS4-4 with 5' and 3' ends for cloning into pET-28b(+) by HiFi Assembly

UP0386	Hw ESS1 5'-F	GCGGCCTGGTGCCGCGCGGCAGCCATATGGATCAGGCGACAGGAC	Amplify Hw ESS1 from pJGS4-4 with 5' and 3' ends for cloning into pET-28b(+) by HiFi Assembly
UP0387	Hw ESS1 3'-R	AGCTTGTCGACGGAGCTCGAATTCGGATCCTTACTCGAGCCGCTCGATAATATGC	Amplify Hw ESS1 from pJGS4-4 with 5' and 3' ends for cloning into pET-28b(+) by HiFi Assembly
UP0388	Wi ESS1 5'-F	GCGGCCTGGTGCCGCGCGGCAGCCATATGACGTGGACGATTAAATTCTCAAACTCACGC	Amplify Wi ESS1 from pJGS4-4 with 5' and 3' ends for cloning into pET-28b(+) by HiFi Assembly
UP0389	Wi ESS1 3'-R	AGCTTGTCGACGGAGCTCGAATTCGGATCCTCACTCCAATCTTAATATCAAGTGGACACC	Amplify Wi ESS1 from pJGS4-4 with 5' and 3' ends for cloning into pET-28b(+) by HiFi Assembly
UP0390	Dc ESS1 5'-F	GCGGCCTGGTGCCGCGCGGCAGCCATATGTCAGGCGCAGCACCCAC	Amplify Dc ESS1 from pJGS4-4 with 5' and 3' ends for cloning into pET-28b(+) by HiFi Assembly
UP0391	Dc ESS1 3'-R	AGCTTGTCGACGGAGCTCGAATTCGGATCCCTATCCGGTTCGCAGTATGAC	Amplify Dc ESS1 from pJGS4-4 with 5' and 3' ends for cloning into pET-28b(+) by HiFi Assembly
UP0392	Nv ESS1 5'-F	GCGGCCTGGTGCCGCGCGGCAGCCATATGTCGAACCCATGGGAAGTCCG	Amplify Nv ESS1 from pJGS4-4 with 5' and 3' ends for cloning into pET-28b(+) by HiFi Assembly
UP0393	Nv ESS1 3'-R	AGCTTGTCGACGGAGCTCGAATTCGGATCCCTAACCCGTCCTCAGGATCAAATGC	Amplify Nv ESS1 from pJGS4-4 with 5' and 3' ends for cloning into pET-28b(+) by HiFi Assembly
UP0402	Dm CTD 5'-F	AATGTCGCCTAGCTGGTCGC	Amplify around Dm CTD
UP0403	Dm CTD 3'-R	CTTTCCCGTTCGGGCGATCG	Amplify around Dm CTD
UP0406	Dc_1 CTD 5'-F	GGCTCGGCAGCCTTCTCGCCG	Amplify around Dc_1 CTD
UP0407	Dc_1 CTD 3'-R	CTAGCTCTTCCAGCTGGGCGACGCCGC	Amplify around Dc_1 CTD
UP0425	Dm CTD 5'-F	GTCTCCTCACCAGGCTATTCGCCAACGAGTCCGAACTACACGG	Amplify *D. melanogaster* RNAPII CTD from pET-SUMO vector, with overhangs to HiFi Assemble it into pFR467
UP0426	Dm CTD 3'-R	GTAATCTCTAGAGGTGTCTTCGCTCTCCTCGAACG	Amplify D. melanogaster RNAPII CTD from pET-SUMO vector, with overhangs to HiFi Assemble it into pFR467
UP0444	Sc CTD 3'-R	GTAATCTCTAGAGGTTCTGGAATTTTCATTTTCATTATGCTTTTGTTCGTCTTGC	Amplify ScCTD from pFR467 to clone back into pFR467 but without a stop codon
UP0445	Ap CTD 3'-R	GTAATCTCTAGAGGTTTGGCCAGGCGAGTTTGGTG	Amplify ApCTD from pFR467 to clone back into pFR467 but without a stop codon
UP0446	Hw_1 CTD 3'-R	GTAATCTCTAGAGGTGTCTTCACGGGGTGAATTAGGC	Amplify HwCTD sequence #1 from pFR467 to clone back into pFR467 but without a stop codon
UP0447	Hw_2 CTD 3'-R	GTAATCTCTAGAGGTGTCTTCACGTGGTGAATTCGGTG	Amplify HwCTD sequence #2 from pFR467 to clone back into pFR467 but without a stop codon
UP0448	Wi CTD 3'-R	GTAATCTCTAGAGGTCGTCTTCCAGCTTGGCCCTG	Amplify WiCTD from pFR467 to clone back into pFR467 but without a stop codon
UP0449	Dc_1 CTD 3'-R	GTAATCTCTAGAGGTGCTCTTCCAGCTGGGCGACG	Amplify DcCTD sequence #1 from pFR467 to clone back into pFR467 but without a stop codon

UP0450	Dc_2 CTD 3'-R	GTAATCTCTAGAGGTACTCTTCCAGCTCGG CGACG	Amplify DcCTD sequence #2 from pFR467 to clone back into pFR467 but without a stop codon
UP0451	Nv CTD 3'-R	GTAATCTCTAGAGGTCCGCCTAGTATGACT TGGC	Amplify NvCTD from pFR467 to clone back into pFR467 but without a stop codon

* Arctic refers to *Aureobasidium pullulans (*Ap*)*, *Hortaea werneckii (*Hw*)* and *Wallemia ichthyophaga (*Wi*)*, although they are not strictly Arctic species, see text.

** Antarctic refers to *Dioszegia cryoxerica* (Dc) and *Naganishia vishniacii* (Nv), species recovered so far only from Antarctica
